# Cheese: mere indulgence or part of a healthy diet?

**DOI:** 10.3389/fnut.2025.1649432

**Published:** 2025-10-03

**Authors:** Ana Eugénio, Rita Ramos, Inês R. Barreto, Raquel Carriço, Joana Marcos, Alexandra Camelo, Christophe Espírito Santo, Inês Brandão

**Affiliations:** ^1^Centro de Apoio Tecnológico Agro Alimentar (CATAA), Castelo Branco, Portugal; ^2^Centre for Functional Ecology, Associate Laboratory TERRA, Department of Life Sciences, University of Coimbra, Coimbra, Portugal; ^3^FitoLab, Laboratory for Phytopathology, Instituto Pedro Nunes, Coimbra, Portugal; ^4^Universidade da Beira Interior, Faculdade de Ciências, Covilhã, Portugal; ^5^Universidade Lusófona, Lisboa, Portugal

**Keywords:** cheese matrix, cardiometabolic health, fermented dairy, bioactive compounds, cheese microbiota, saturated fat, Protected Designation of Origin (PDO), randomized controlled trials

## Abstract

Cheese is a widely consumed fermented dairy product with a long history of human consumption dating back several millennia, which justifies a brief historical introduction in this review. Beyond its cultural and gastronomic relevance, cheese presents a complex nutritional and microbial matrix that may confer neutral or even beneficial effects on cardiometabolic health, despite concerns related to its saturated fat and sodium content. This review first explores the key components of the cheese matrix and several mechanisms potentially involved in its metabolic impact, including the presence of polar lipids, the production of short-chain fatty acids (SCFAs) and alpha-linolenic acid (ALA) by the cheese microbiota, and the high calcium content that may reduce fat absorption, among others. Additional bioactive compounds formed during fermentation, such as angiotensin-converting enzyme (ACE)-inhibitory peptides, are also discussed for their potential health effects. We also include a comprehensive survey of most European Protected Designation of Origin (PDO) cheeses and their microbiota. Finally, to assess the most recent evidence in human health, we analyzed a sample of studies published on PubMed in the last 2.5 years, including observational studies and randomized controlled trials. This selection highlights the latest research trends and supports a growing body of evidence suggesting that cheese, particularly in its intact matrix form, is not associated with adverse cardiometabolic outcomes—and may even exert protective effects. These findings call for more robust, long-term trials to confirm causality and support updated dietary guidance.

## 1 Introduction

Advances in nutrition science are making clear that the focus on calories and isolated nutrients, which were the basis for the creation of conventional dietary guidelines during the 20th century (1, 2), is no longer sufficient to predict the impact of food on health, especially when it comes to the prevention of chronic and complex cardiometabolic diseases, such as type 2 diabetes mellitus (T2DM) or obesity (3–6). More in-depth studies of foods as a whole, i.e. on their complex matrices composed of nutrients, minerals, bioactive compounds, and other factors, such as prebiotics and probiotics, become inevitable to understand the real impact of foods on health and disease.

A relevant example of this is cheese, a widely produced and consumed fermented dairy product, that is a major source of saturated fat and salt, two components that are traditionally linked to cardiometabolic diseases (7–9). Due to this, cheese has been a regular target to cut down from diet, when considering the traditional guidelines.

However, an increasing body of scientific evidence has been showing that there may not be a strong correlation between cheese consumption and disease (10–12). Various studies during the last decade have shown that high consumption of fermented dairy products, namely cheese, is not consistently related to cardiovascular disease (CVD) or even to mortality risk, even though these foods are high in saturated fat (13–15). In fact, cheese nutrients may possibly work in concert to lower markers of cardiovascular risk, compared to other dairy products when matched for fat content (16, 17), and have other beneficial effects on human health (18, 19). Nevertheless, a detailed mechanistic study of how the cheese matrix influences health outcomes is still needed.

The present review, after a brief historical overview—justified by the millennia-long presence of cheese in the human diet—aims to explore the composition of the cheese matrix in detail and its potential implications for cardiometabolic health. We examine its key nutritional components, including proteins, lipids, carbohydrates (oligosaccharides), minerals (macro and microelements), and bioactive components (peptides, organic acids, vitamins, and exopolysaccharides). Finally, the diverse cheese microbiota is also thoroughly explored.

In addition, we address the process of milk pasteurization and provide a comparison between pasteurized and raw milk cheeses. As highlighted in multiple studies in recent years, cheese – despite its high saturated fat and sodium content – is increasingly reported in observational studies and randomized controlled trials to have neutral or even beneficial associations with cardiometabolic outcomes. These findings are often attributed to the “dairy matrix effect”, in which the interactions between nutrients, minerals such as calcium, bioactive peptides, and the fermentation-derived microbiota may modulate lipid digestion, sodium effects, and other metabolic responses.

As part of this work, a detailed survey of the majority of European Protected Designation of Origin (PDO) cheeses is also carried out. Focusing on PDO cheeses allows a more reliable characterization of their microbiota compared to non-PDO cheeses, whose production methods and microbial compositions are often more variable and less well documented. Their traditional starter cultures and dominant microbial taxa are rigorously described in the **Supplementary material**. This microbial characterization supports a broader understanding of how traditional cheese-making practices shape both nutritional properties and health effects.

Finally, to explore these health associations in more detail, we analyzed the most recent human studies published in PubMed over the past 2.5 years (starting on January 2023), encompassing both observational studies and randomized controlled trials. Focusing on this recent period to update the field without duplicating the evidence already synthesized in recent high-quality meta-analyses, as illustrated by Zhang et al. (20), Pradeilles et al. (21) and Al Slurink et al. (22). This approach provides a comprehensive perspective on how the cheese matrix functions and how it may influence cardiometabolic health.

## 2 Brief historical context

Cheese, either fresh or matured, is an easily digestible product obtained from the coagulation of milk. In short, raw or pasteurized milk is heated and a coagulant, usually rennet, is added to the milk, causing it to curdle and separate between a solid phase, the curds, and a liquid phase, the whey. The whey is drained off and the curds are pressed to remove additional whey and to shape the cheese. Salt is then added by mixing, brining or rubbing. Afterwards, the cheese is aged for varying periods, depending on cheese type, which allows it to develop flavor and texture, by the action of bacteria, fungi and enzymes (23, 24).

Cheeses are differentiated according to milk source (cow, sheep, goat, buffalo, yak, llama, moose, etc.) and other factors such as their manufacturing process (use of animal or plant rennet, or sour milk), consistency (extra-hard, hard, semi-hard, semi-soft, soft, fresh), fat content (double cream, cream, full fat, three-quarters fat, half fat, quarter fat), fermentation type (lactic acid, lactic and propionic acid, butyric acid), microbiota, and physical appearance (hard, soft, with smear, molds) (10, 25, 26).

Cheese has been produced and consumed throughout the world since ancient times. The art of cheese-making dates back to the early Neolithic period during the 6th millennium B.C., with the domestication of sheep and goats, and in agreement with the abundant milk residues in ancient ceramic vessels found in Poland (27–30). Presumably, the process of cheese-making was discovered unintentionally by storing milk in containers made from the stomach of unweaned baby animals, which contained a coagulation enzyme, rennin (chymosin), turning milk into curd and whey (31, 32). In fact, this technique was maintained until the beginning of the 20th century, in some Mediterranean regions (33, 34). Cheese-making was undoubtedly a major achievement for ancient farmers, as it allowed the preservation of milk in a non-perishable and transportable way. Furthermore, the processing of milk made it more digestible for consumption, especially for people that are lactose-intolerant (10, 35, 36).

Romans further mastered the art of cheese-making by developing ripening techniques that would lead to different flavors and characteristics. This expertise was spread throughout the Roman Empire and cheese became an everyday foodstuff (33). During the Middle Ages, European monks improved ripening and aging techniques and developed several varieties of cheese that are still marketed at the present time (33). Later, during the Renaissance period, cheese declined in popularity, most likely due to the poor hygiene conditions found at the time in traditional cheese-making farms and during commercialization (29, 37). This product regained popularity in the 19th century, when cheese production was introduced in factories (33, 38, 39).

A new era for food manufacturing began in the 1860s, with the scientific discovery of pasteurization by Louis Pasteur (40). As milk provides a favorable environment for microbial growth, the adoption of pasteurization for this product was crucial and widely spread in developed countries, allowing for a more controlled and safer cheese production at the industrial scale (41). The World Wars and the Great Depression have further driven innovation in cheese-making techniques, in order to make products that were cheaper, more durable and with longer shelf-life (42).

Nowadays, there is a large diversity of cheese, with hundreds of varieties described (25), based on the type of milk, heat treatment, coagulation method, curd preparation, fat content, moisture, and ripening time. It can also be further flavored with herbs, spices, and smoke. Cheese is produced all over the world. For the 2024/2025 marketing year, the European Union has been the largest producer, with an estimated 10.7 million metric tons (about 47 % of global output) and also the largest consumer, accounting for roughly 43 % of world cheese consumption, a pattern that parallels its production. In terms of production, the United States follows with around 6.46 million metric tons (29 % of global production). Other significant producers include Russia (≈1.16 million metric tons) and Brazil (≈0.78 million metric tons) (43).

The impressive variety and prevalent consumption all over the world demonstrates the importance of studying the cheese matrix and the effects of this dairy product on health.

## 3 Cheese matrix: a complex structure with nutrients, bioactive components and microbiota

### 3.1 Nutritional components

Dairy product matrices differ considerably from each other, which explains why dairy foods vary in the nutrition and health outcomes they provide, due to the distinct bioavailability of nutrients and bioactive compounds (44). Nutritional values alone are insufficient to predict the effects of dairy products on health, and thus, physical-chemical and biological properties, along with the possible interactions between all components must be fully explored (17, 45).

In fact, the unique structure and physical-chemical features of each food determine how it is digested and, consequently, the absorption of the nutrients and other components contained in the food (46, 47). For example, several studies have reported total and LDL cholesterol to raise significantly after intervention periods of butter consumption, when compared to the same period of cheese consumption (44, 48, 49). Regarding the dairy matrix effect in circulating postprandial amino acid levels, one study revealed these levels increased more quickly and to higher levels, but also decreased much more rapidly on consumption of stirred yogurt compared to cheese, with the latter showing a much more gradual release (50). The more gradual and sustained release into the intestine is assumed to be beneficial for a better degradation of the food matrix, promoting the absorption of matrix components like calcium (51).

Cheese matrices are formed by complex networks that include macro and micronutrients, namely hydrated proteins enclosing scattered fat globules, bioactive peptides, SCFAs, conjugated linoleic acids (CLAs), and other factors, such as minerals like calcium, zinc, phosphorus and magnesium, vitamins, antioxidants, prebiotics, and probiotics (52, 53).

The amount of each major and minor component of these matrices is variable, according to the type of cheese. For example, Parmesan and Gruyère are hard cheeses, containing higher levels of protein, fat, calcium and salt, but lower amount of moisture, contrasting with Fromage Frais and Cottage, which are soft cheeses containing higher moisture, but lower amounts of these other components (17, 54–56).

The structure of the cheese matrix is also dependent on the type of cheese. For example, Cream cheese is formed by compact fat and protein aggregates with large spaces filled with whey protein (57, 58), Cheddar cheese is characterized by large irregular fat pools (57, 59), Mozzarella has a fibrous and highly oriented structure, capable to stretch, with fat globules and water organized between protein fibers, preventing their coalescence, and creating columns in the direction of stretching (60, 61).

The components of the cheese matrix and their structural organization can significantly impact the matrix's breakdown during mastication and digestion and therefore, change the way nutrients and bioactive components are released and, ultimately, absorbed. As highlighted by O'Connor et al. (62), even though intervention diets were matched for fat, protein, and calcium, the group consuming the melted cheese showed worse metabolic outcomes compared to those consuming unmelted cheese. This suggests that the intact cheese matrix may modulate nutrient release and digestion, possibly affecting lipid metabolism. For instance, lipids in the unmelted cheese matrix may bind with calcium and form calcium soaps, therefore lowering fat absorption (62).

Other biological mechanisms may help explain the association between fermented dairy products and cardiometabolic health, such as the microbiota found in fermented milk and cheese. For instance, cheese bacteria are believed to produce SCFAs and ferment indigestible carbohydrates, which can inhibit cholesterol synthesis and lower blood cholesterol levels. Additionally, bacteria in the large intestine can bind cholesterol to bile acids, forming cholesterol-bile acid complexes that are excreted in the feces. This reduces bile acid circulation, which in turn limits cholesterol uptake into the liver.

#### 3.1.1 Proteins

Milk proteins are an important component of the cheese matrix, with amounts varying from about 4% in Cream cheese to around 40% in Parmesan (55, 58). The two proteins in milk are casein and whey, which are high-quality proteins containing all essential amino acids (63–65). The relative amount of these two proteins in milk varies according to species: while there is an 80:20 casein to whey ratio for cow, sheep, goat and buffalo milk, this ratio is about 40:60 in human and 50:50 in quine milk (64).

The manufacture of natural cheese involves the coagulation of casein micelles, that separate from the whey liquid phase. Whey can be washed and dried into powder, to be used in the production of other food products (23). Most cheeses are casein-based, but some cheeses, such as ricotta and mizithra, are primarily made with whey (66). Casein-cheeses and whey-cheeses are considered different food categories in the *Codex Alimentarius* (CODEX STAN 283-1978 and 284-1971, respectively). Besides natural cheeses, processed cheeses are also commercialized, and they are made by blending natural cheeses with emulsifying salts and other dairy and nondairy ingredients (67).

As mentioned, casein and whey are high-quality proteins that are also easily digested and absorbed, albeit at a different speed. Casein provides an efficient nutrient supply, by promoting a slow and prolonged postprandial release of amino acids in the blood stream (68, 69). This slow release of amino acids promotes muscle growth and reduces protein breakdown, enhancing long-term muscle mass (68, 70). On the other hand, whey is digested more quickly than casein, and provides a fast source of available nutrients and amino acids to the body (68). Both casein and whey are also sources of leucine, which is an essential amino acid that induces the synthesis of muscle proteins (71, 72).

Due to these proprieties, casein and whey have been purposely included in diets or taken as supplements, especially by athletes and bodybuilders, and also by older people, to maintain or increase lean body mass, with a growing number of studies supporting the beneficial effects of these proteins (73–77). For example, one study observed the superior increase of muscle size in young-adults after a 10-week resistance training program supplemented with whey protein, when compared to the same training supplemented with leucine-matched collagen peptide (78), and another study showed that the introduction of ricotta cheese in the diet of older people attenuated the loss of muscle strength (79). Nevertheless, research has also been cautioning against taking these proteins as supplements and the risk of excessive intake, especially for non-athletes. For example, one study showed that milk protein intake above the recommended dietary allowance did not increase body mass in functionally limited older men (80), and some studies have been associating the excessive consumption of these proteins with kidney and liver damage, acne and modification of the microbiota (81–84).

It is interesting to note that the digestion of these proteins can be affected by cheese manufacture. For example, pasteurization and other heat treatments applied to milk have been suggested to improve protein digestion rate in the human gastrointestinal tract (85, 86). Also, allergies to caseins are common, especially concerning β-caseins: the genetic variant A1, found in milk from certain breeds like Holstein cows, is associated with the production of a peptide called beta-casomorphin-7 (BCM-7) and has been shown to cause slow digestion and inflammation. A2 variant, from breeds like Guernsey and Jersey cows, seems to be less likely to cause gastrointestinal issues. More studies on the differences between β-Casein A1 and A2 are still necessary before recommendations on consumption are made (87, 88).

All these studies highlight that a systematic and more comprehensive research on the effects of casein and whey in human health is still necessary, especially in the scope of regulation and education about their safe intake through food and supplements.

#### 3.1.2 Lipids

The lipid fraction of milk is contained in fat globules and mainly composed of triglycerides (TGs) (~98% of total lipids), but also by fatty acids, acylglycerols, phospholipids, cholesterol and other lipophilic molecules, like vitamins (A, D, E and K) and carotenoids (β-carotene) (89, 90). These globules are naturally enveloped by the milk fat globule membrane (MFGM), a complex trilayer with biologically active functions and composed of ~ 70% proteins and milk polar lipids such as phospholipids and sphingolipids (91–93).

Milk polar lipids from the MFGM have been associated with cardiometabolic benefits, particularly through the reduction of plasma and hepatic hyperlipidemia. These effects are largely attributed to their ability to reduce intestinal cholesterol absorption by multiple mechanisms: they promote intraluminal emulsification, decrease cholesterol solubility in mixed micelles, and inhibit fat digestion by binding of sphingomyelin to pancreatic colipase, thereby reducing pancreatic lipase activity (92, 94, 95). Despite the great focus on the beneficial effects of polar lipids with several evidence in the literature, the protein and glycoprotein fractions of MFGM should not be ignored from a nutritional and bioactive perspective (96).

Processing milk substantially changes the MFGM structure. During cheese production, treatments such as homogenization or pasteurization can disrupt the MFGM and approximately 20% of the phospholipids are retained in the whey (97, 98). The coagulation of milk forms a semi-solid milk gel with milk fat globules entrapped within a casein protein network, and organized as either individual fat globules, aggregated, coalesced or elongated globules, depending on cheese manufacturing processes (99, 100).

Homogenized milk, often used in the production of soft cheeses, contains smaller fat globules that enhance moisture retention. In contrast, unhomogenized milk is typically associated with hard and semi-hard cheese varieties (101–104). The digestion of these fat globules is dependent on both their structural organization (105) and also on the extent of the cheese matrix disintegration during digestion, which varies according to cheese type (106, 107). Fat digestion has also been shown to vary according to the degree of lipid distribution within cheese matrices: cheese manufactured with homogenized milk, as the case of cream and some blue mold cheeses, was shown to have a faster released of free fatty acid from the cheese matrix (85, 100, 108). Furthermore, emulsification can also impact fat digestion (109, 110).

In conclusion, the lipid component of cheese plays a multifaceted role in its structure, sensory characteristics, and potential health implications. Although traditionally viewed as a source of saturated fat, emerging research emphasizes the importance of considering the cheese matrix, the presence of bioactive lipids, and the complexity of lipid digestion when evaluating its health impact.

#### 3.1.3 Carbohydrates

Cheese has a relatively low carbohydrate content when compared to other foods, and is predominantly comprised of lactose, a disaccharide made up of D-galactose bound to D-glucose (111, 112).

Lactose is the primary carbohydrate in milk, comprising about 4.8–5.0 mg/100 mL of cow's milk (113), and plays a significant role in the initial stages of cheese production (25). However, during this process, most of the lactose present is removed along with whey, and the residual lactose in the curds is fermented by lactic acid bacteria (LAB), further reducing its content (114).

Thus, the amount of lactose in most cheese types is very small, especially in aged cheeses when compared to fresh cheeses, due to the longer fermentation period that allows more time for lactose to be broken down—for example, Cheddar, Brie, and Camembert are aged cheeses that contain only trace amounts of lactose (10). Because of this, individuals with lactose intolerance are still able to consume most cheeses, without experiencing significant symptoms (10, 35, 115). Moreover, some studies suggest that cheese consumption may even have a protective effect on gut microbiota by providing LAB with probiotic properties, which may further aid in lactose metabolism (116).

However, fresh and unripened cheeses such as Ricotta, Cottage cheese, and Cream cheese may retain slightly higher levels of lactose and could pose a risk for more sensitive individuals when compared to hard cheeses like cheddar and gruyere or even aged ones like Parmigiano Reggiano and Grana Padano (117, 118). Therefore, it is important for lactose-intolerant consumers to distinguish between cheese types and select those that are naturally low in lactose or specifically labeled as lactose-free.

#### 3.1.4 Minerals

Cheese is an important source of several essential minerals, with calcium being the most prominent. The addition of calcium reduces the rennet coagulation time of milk by neutralizing the negatively charged residues on casein, which enhances the aggregation of renneted micelles. Also, the high calcium content of cheese influences the dairy fat matrix, as the interaction between milk calcium and caseins affects the formation of the protein network within which the MFGM is embedded (104). As previously mentioned, the formation of calcium soaps interferes with lipid digestion by reducing fat absorption (62). This mineral impacts cheese texture as low concentrations of calcium contribute to increased gel firmness (119), and its amount can significantly vary depending on cheese type: Cheddar, Gruyère, and especially Parmesan contains some of the highest amount of calcium among cheeses (about 7 to 12 milligram of calcium per gram of cheese “as consumed”; “FoodData Central” (120).

Calcium bioavailability in cheese is generally high due to its integration within the casein matrix, particularly in the form of caseinophosphopeptides (CPPs), which are generated during gastrointestinal digestion. These phosphorylated peptides have a high affinity for minerals such as calcium, helping to maintain their solubility and promoting passive absorption in the distal small intestine (121, 122). Dietary calcium is vital for the development and maintenance of bones and teeth (123). It has also been associated with muscle function (124, 125), and weight management (126), as well as playing a role in nerve transmission (127, 128), blood pressure (129), and the regulation of hormones and enzymes (130). Phosphorus is another mineral present in cheese, often in a balanced ratio with calcium (131). It contributes to skeletal integrity, nucleic acid and protein synthesis, and oxygen transport (132).

Although calcium and phosphorus are crucial for skeletal health, excessive intake of these minerals, especially when taken as supplements, has raised concerns about their potential negative effects on health. Research has suggested that dietary calcium and phosphorus intake should primarily come from food sources, such as cheese, to avoid the risks associated with over-supplementation (133–137).

Furthermore, cheese also presents smaller amounts of magnesium, potassium, zinc, cooper, and selenium (138). These trace elements play important roles in various metabolic processes, such as enzyme function, immune response, and antioxidant activity.

And finally, cheese contains a relatively high sodium (salt) content, which is an important consideration for those monitoring its intake for health reasons (139).

Aged cheeses, such as Parmesan, Cheddar, and Roquefort, contain especially high sodium concentrations due to the aging process and the salt used in brining: it is added to enhance flavor, acts as a preservative by inhibiting the growth of undesirable bacteria and mold, and plays a key role in the overall texture and maturation of cheeses (23, 140).

Sodium is essential for maintaining fluid balance, nerve function, and muscle contraction (141). However, high sodium intake is mostly associated with increased risk of hypertension and CVD (142). The World Health Organization recommends limiting sodium intake to < 2 grams per day to reduce health risks [“Sodium reduction” (143)]. Given that cheese can be a significant source of dietary sodium, those with hypertension or other cardiovascular conditions should be mindful of their cheese consumption: some strategies can include controlling portion size or choosing reduced-sodium versions of some cheeses (139, 144–146).

Curiously, despite its sodium content, some studies have reported an antihypertensive effect of cheese (147, 148). For instance, a randomized, double-blind, placebo-controlled pilot study by Crippa et al. found that daily consumption of 30 g of Grana Padano P.D.O cheese significantly reduced blood pressure in mild to moderate hypertensive subjects. This antihypertensive effect may be partly explained by the presence of angiotensin-I-converting enzyme (ACE)-inhibiting peptides naturally released during the cheese's long ripening process, which can help counteract the hypertensive impact of sodium. These findings suggest that certain aged cheeses like Grana Padano might offer cardiovascular benefits beyond their mineral composition, although moderation remains important for individuals sensitive to sodium (148).

### 3.2 Bioactive components

#### 3.2.1 Peptides

Bioactive peptides are biological molecules, with fewer than 50 amino acids linked together by peptide bonds, that are derived from food proteins and become activated when these proteins are cleaved either by enzymes or by microbial fermentation (149–151). They have high tissue affinity, do not accumulate inside organisms, and have important beneficial effects in human health, for which they have been a target of an increasing number of studies (152–154). For example, these physiologically active peptides have been shown to possess anti-inflammatory (155–158), antioxidant (159–161), anticancer (162–164), and immunomodulating (165, 166) proprieties.

In cheese, bioactive peptides are derived from casein and whey proteins, and their concentration is dependent on cheese manufacture, including the starter bacterial culture, processing conditions (namely, milk heat treatment), and ripening stage (167, 168).

The beneficial effects of consuming cheese for their bioactive peptides have been under study, with research showing that some cheeses contain peptides with functional antihypertensive, antimicrobial, antioxidant, anticarcinogenic, opioid, and zinc-binding properties (169). For example, it was shown that the consumption of Domiati, Edam, and especially Gouda cheeses could exert antihypertensive effects, due to the presence of the ACE-inhibiting peptides, namely the tripeptides IPP (Ile-Pro-Pro) and VPP (Val-Pro-Pro), in their matrices (170, 171). It was also observed antibacterial proprieties derived from bioactive peptides, against both gram-positive and gram-negative bacterial species, in the Italian cheeses Pecorino Romano, Canestrato Pugliese, Crescenza, and Caprino del Piemonte (172). Although peptides present in cheese exhibit significant bioactive potential, their clinical efficacy depends on the ability to survive within the gastrointestinal tract, the systemic bioavailability, and interactions with the gut microbiota (173).

#### 3.2.2 Organic acids

Dairy fat is composed of nearly 400 different fatty acids, including saturated, monounsaturated (MUFAs), polyunsaturated (PUFAs), trans-fatty acids, and branched-chain fatty acids, each with biological significance (174). Among these, saturated fatty acids are the most abundant, accounting for ~60–70% of the total fatty acid content (10, 89).

Cheeses have a variable amount of fat content, varying from < 8% (~4 g of fat in Cottage and ~7 g of fat in Fromage frais, per 100 g of dry matter, for example) to around 35% (~34 g of fat in Cheddar and ~36 g of fat in Roquefort, per 100 g of dry matter, for example). It is an important component of cheese matrices, largely contributing to flavor and texture (175).

Beyond their structural and sensory roles, certain unsaturated fatty acids present in cheese fat have garnered attention for their potential health benefits. Oleic acid, the predominant MUFA in cheese, has been linked to cardioprotective effects, including improved lipid metabolism, enhanced endothelial function, and anti-inflammatory properties (176, 177).

CLAs, a group of linoleic acid isomers naturally found in ruminant-derived dairy fat, has been particularly noted for its anticarcinogenic, antiadipogenic, antiatherogenic, and immunomodulatory activities (178, 179). The CLAs content in cheese is highly influenced by dairy animal diet, with pasture-based feeding systems significantly increasing CLAs levels (179, 180). Other factors, such as the composition of the cheese microbiota, also influence CLA levels. Certain probiotic bacteria—*Lactiplantibacillus plantarum, Lactobacillus acidophilus, Lacticaseibacillus casei*, and *Bifidobacterium lactis*—can increase CLA content by converting linoleic acid during the ripening process. Regarding milk fat sources, CLA levels tend to follow this ascending order: caprine < bovine < ovine milk (181).

Additionally, PUFAs such as ALA and vaccenic acid also contribute to the potential cardiometabolic benefits of dairy fat (182).

#### 3.2.3 Vitamins

There are several vitamins present in cheese matrices, namely vitamins A, B2, B12, D, E, and K2.

Vitamin A is important for vision, skin health, and immune function. It is present in cheese in the form of retinol and beta-carotene, and hard cheeses, such as Cheddar and Parmesan, are particularly good sources of this vitamin (183, 184).

Cheese is also rich in different B vitamins. B2 (riboflavin) plays a role in mitochondrial function and the metabolism of fats, drugs, and steroids. It is also important for healthy skin, eyes, and nerve functions (185, 186). B12 (cobalamin) is important for the formation of red blood cells, DNA synthesis, and neurological function. Cheese is one of the few non-meat sources of vitamin B12 (187, 188).

Though not a major source, cheese can also contain smaller amounts of vitamin E, an important antioxidant that inhibits the process of lipid peroxidation (189), and vitamin D, significant for calcium absorption and bone health (190, 191).

Vitamin K2 (menaquinone) is present in particularly high amounts in hard and aged cheeses, such as Gouda and Edam. This vitamin contributes to cardiovascular health by preventing and potentially reversing vascular calcification, supports bone integrity by enhancing the γ-carboxylation of osteocalcin and increasing osteoprotegerin levels, and helps preserve cognitive function by activating proteins such as Gas6 and protein S, as well as promoting the synthesis of sphingolipids (192–196).

While cheese is naturally rich in vitamins, there has been a growing trend in fortifying cheese with additional vitamins, particularly A and D, to further enhance its nutritional value. This fortification process aims to address common nutritional deficiencies and improve public health by making these vitamins more accessible through a product that is widely consumed (197).

#### 3.2.4 Exopolysaccharides

Exopolysaccharides (EPS) are produced by microorganisms, including bacteria, fungi and algae, and are involved in the formation of extracellular biofilms that provide protection against potential environmental stressors, such as temperature and antibiotics (198–201). In cheese, EPS can play roles in shaping the microstructure, texture, and functionality of the cheese matrix (202, 203).

EPS-producing strains of lactic acid bacteria (LAB) commonly found in cheese, such as *Lactococcus, Lactiplantibacillus, Leuconostoc*, and *Streptococcus*, have been shown to interact with casein micelles and fat globules, increasing moisture retention (204, 205) and reducing syneresis (206, 207) within the matrix, contributing to maintain or improve cheese texture and cooking properties (206–208). For example, the presence of EPS-producing *Streptococcus thermophilus* has been shown to make the Karish cheese more deformable and softer (209) a EPS-producing *Lactococcus lactis* ssp. *cremoris* strain has increased yield by around 8% and moisture content by around 9.5% in a half-fat cheddar (208), and a mixed starter culture containing EPS-producing *Lactobacillus delbrueckii* subsp. *bulgaricus* and *Streptococcus thermophilus* has resulted in a higher moisture content and meltability of low-fat Mozzarella cheese (210).

Furthermore, EPS can also impact biochemical processes during cheese ripening, including influencing proteolytic activity, thereby affecting flavors and maturation rates. For instance, the proteolysis in reduced-fat Cheddar cheese was shown to increase in the presence of a EPS-producing *Lactococcus lactis* ssp. *cremoris* (JFR1) strain (203).

Due to these effects, EPS-producing bacteria can serve as natural additives to improve moisture, texture, melting, and sensory properties of low and reduced-fat cheeses, promoting the consumption of healthier cheese variants (200, 207, 208, 211). These benefits add to the health-promoting potential that EPS are being shown to exhibit, which include wound healing (212, 213), drug delivery (214, 215), immunomodulation (216–218), antimicrobial (219, 220), and anticancer (220, 221) properties.

## 4 Cheese microbiota

Cheese contains a diverse microbial community that is significantly influenced by manufacturing, particularly ripening conditions, and hence contributes importantly to quality, safety, and physical-chemical properties (24, 222).

The cheese microbiome varies greatly depending on the type of cheese, the environment and processing conditions, pasteurization methods and respective temperature, and ripening conditions (see Table 1, Supplementary Table S1). Microorganisms play an active role in determining cheese composition and influence the flavor profile through the production of volatile compounds (26, 222, 223). Microbial diversity is influenced by the origin of the milk, with cow's milk appearing to be more diverse than milk from goats and sheep and can range within the cheese from the core to the surface (222, 223). Different microbiological compositions can be found in the rind and core of cheese. This is partly due to variations in oxygen supply throughout the cheese (223). The cheese rind is an aerobic environment and is constantly exposed to possible contamination by external sources, so the presence of oxygen on the surface of the cheese permits the growth of aerobic organisms, which are unable to grow more profoundly, as there is less oxygen availability (26, 223). During ripening, the core becomes an anaerobic environment, making it less susceptible to external contamination (26). These microorganisms can play important roles in fermentation, aging, texture and flavor of cheese, as well as acting as probiotics, providing health benefits.

**Table 1:** Short-version of the PDO cheeses microbiota.

**PDO cheese**	**Country of origin**	**Milk treatment**	**Starter culture**	**Dominant taxa**	**References**
West country farmhouse cheddar	United Kingdom	Raw or Pasteurized	*Lactococcus (Lc.) lactis* subsp *cremosis* *Lc. lactis* subsp *lactis*	*Streptococcus (Str.)* *Lactococcus* *Lactobacillus (Lb.)*	(266–269)
Blue Stilton cheese	United Kingdom	Pasteurized	*Lc. lactis* *Penicillium (P.) roqueforti* spores	*Lb. plantarum* *Levilactobacillus (Lv.) brevis Debaryomyces (D.) hansenii* *Kluyveromyces (K.) lactis* *Yarrowia (Y.) lipolytica* *Trichosporon (T.) ovoides* *Lc. lactis*, *Enterococcus (E.) faecalis*, *Lb. curvatus*, *Leuconostoc (Leuc.) mesenteroides* *Staphylococcus spp*. *Staphylococcus (S.) equorum* *P. roqueforti* *Candida (C.) catenulata*	(232, 270–273)
Noord-Hollandse Gouda	Netherlands	Pasteurized	*Lc. lactis subsp cremosis* *Lc. lactis subsp lactis*	*Lc. lactis subsp. cremoris* *Lc. lactis* *Tetragenococcus (Tet.) halophilus*, *Loigolactobacillus (Lgb.) rennini* *Lc. laudensis* *Leuc. pseudomesenteroides* *Lc. cremoris* *Lacticaseibacillus (Lcb.) paracasei* *Leuc. mesenteroides* *S. equorum* *Tet. halophilus*	(274–277)
Halloumi	Eastern Mediterranean (Cyprus)	Raw or Pasteurized	No	*Lb. manihotivorans* *Lb. alimentarius* *Lv. brevis* *Lb. parakefiri* *Marinilactibacillus psychrotolerans* *Lb. cypricasei*	(278–281)
Mozzarella di Bufala Campana PDO	Italy	Raw, thermalised or Pasteurized	Natural whey starter culture: Str. thermophilus Lb. delbrueckii Lb. helveticus Lc. lactis	*Str. thermophilus* *Lb. helveticus* *Lb. delbrueckii subsp. delbrueckii* *Lb. delbrueckii subsp. bulgaricus* *Str. salivarius* *Lb. delbrueckii*	(282–285)
Parmigiano Reggiano	Italy	Raw	Natural whey starter: *Lb. helveticus Lb. delbrueckii ssp. lactis* *Lb. delbrueckii ssp. bulgaricus* *Lb. rhamnosus*	*Lb. helveticus* *Lb. delbrueckii* *Lacticaseibacillus group* *Lb. fermentum* *Str. thermophilus* *Lb. crispatus* *Lcb. casei* *Lcb. paracasei ssp. paracasei* *Lcb. paracasei ssp. tolerans* *Lv. brevis* *Lb. rhamnosus* *Lb. curvatus* *Pediococcus (Ped). acidilactici* *Lb. delbrueckii subsp. lactis* *Lb. delbrueckii subsp. bulgaricus*	(286, 287)
Gorgonzola (Blue cheese)	Italy	Raw or Pasteurized	*St. thermophilus*, *Lb. delbrueckii*, *Lactococcus* sp. *P. glaucum*, *P. Roqueforti*	*P. roqueforti*, *S. equorum*, *Brevibacterium (B.) linens* *Corynebacterium flavescens* *E. faecium* *Carnobacterium* *S. saprophyticus* (surface) *Aspergillus flavus* *Cladosporium (Cla.) cladosporioides* *Cordycepts farinosa* *D. hansenii* *Fusicolla aquaeductuum* *Mucor (Mu.) circinelloides* *Mu. fuscus* *Mu. lanceolatus* *Mucor sp*. *P. atrosanguineum* *P. camemberti* *P. commune* *Penicillium sp*. *Sporobolomyces deformans* *Y. lipolytica* *Saccharomyces (Sch) cerevisiae var. boulardii* *Arthrobacter sp*. *Carnobacterium sp*. *Staphylococcus sp*. *B. linens* *Phychrobacterium sp*. *Cobetia sp*. *S. lentus*	(232, 288–290)
Pecorino Romano	Italy	Raw	Natural whey starter culture: *Str. thermophilus* *Lb. delbrueckii* subsp. *lactis*, *Lb. helveticus*	*D. hansenii* *K. marxianus* *Rhodotorula spp*. *Sch. cerevisiae*	(234, 291, 292)
Asiago	Italy	Raw	Thermophilic starter culture	*Lc. lactis subsp. lactis* *Lcb. paracasei/rhamnosus* *Enterococcus sp*. *Lactiplantibacillus (Lpb.) plantarum* *Lb. gallinarum* *Lb. delbrueckii* *Limosilactobacillus (Lim.) fermentum* *Str. thermophilus*	(293)
Grana Padano	Italy	Raw	Natural whey starter culture: *Lb. delbrueckii subsp lactis* *Lim. fermentum* *Lactobacilllus helveticus* *Str. thermophilus*	*Lb. delbrueckii* *Lcb. rhamnosus* *Lcb. casei* *Lim. fermentum* *Lc. raffinolactis*, *Lb. helveticus* *Str. thermophilus* *Lc. lactis*	(294–297)
Provolone del Monaco	Italy	Raw	No	*Lcb. casei* *Lcb. paracasei* *Str. macedonicus* *E. faecalis*	(298)
Feta	Greece	Raw or Pasteurized	*Str. thermophilus* *Lb. delbrueckii subsp. bulgaricus*	*Lb. plantarum* *Lv. brevis* *Lcb. paracasei* *Lb. rhamnosus* *Lb. paraplantarum* *Lb. curvatus* *E. faecalis* *E. faecium* *E. durans* *E. malodoratus* *Str. salivarius subsp. thermophilus* *Lb. coryniformis* *Lb. fermentum* *K. lactis* *Pichia (Pich.) membranifaciens* *C. krisii/zeylanoides* *Pich. fermentans* *Lc. piscium* *Lc. raffinolactis* *Lcb. zeae* *Str. uberis*	(299–302)
Brie (de Meaux and de Melun)	France	Raw	*Lc. lactis subsp. lactis* *Lc. lactis subsp. cremoris* *Leuc. mesenteroides subsp. cremoris*	*P. candidum?* *Brachybacterium* *Micrococcaceae* *Carnobacterium* *Staphylococcus* *Enterococcus* *Hafnia-Obesumbacterium* *Psychrobacter* *Brevibacterium* *Glutamicibacter* *Leucobacter (Brie de Meaux)* *Pediococcus (Brie de Melun)* *Dipodascus* textitPenicillium *Scopulariopsis*	(303–305)
Camembert de Normandie	France	Raw	*Lc. lactis subsp. Lactis* *Lc. lactis subsp. cremoris*	*Lc. lactis* *Str. thermophilus* *Leuc. mesenteroides* *Lb. fermentum* *Lb. plantarum* *Lcb. paracasei*	(306–308)
Roquefort (Blue cheese)	France	Raw	*Leuconostoc spp. Lc. lactis subsp cremosis Lc. lactis subsp lactis Lc. lactis subsp lactis biovar diacetylactis Leuc. mesenteroides subsp mesenteroides P. roqueforti*	*P. roqueforti* *Candida* *Debaryomyces* *Galactomyces* *Yarrowia* *D. hansenii (C. famata)* *K. lactis (C. sphaerica)* *Candida spp. (Surface)*	(232, 309, 310)
Comté	France	Raw	*Str. thermophilus* *Lb. helveticus*	*Lb. delbrueckii subsp. Lactis* *Lb. fermentum* *Lcb. paracasei subsp. paracasei* *Lb. rhamnosus*	(311, 312)
Reblochon de Savoie	France	Raw	*Lactic starter culture*	*Geotrichum (Geo.) candidum* *C. famata* *D. hansenii* *Lb. delbrueckii ssp. bulgaricus* *Str. thermophilus*	(313–315)
Gruyère	Switzerland	Raw	*Lb. helveticus* *Str. thermophilus* *Lb. delbrueckii subsp. lactis*	*Brachybacterium alimentarium* *Brachybacterium tyrofermentans* *Lb. helveticus*	(316–318)
Raclette du Valais	Switzerland	Raw or Pasteurized	*Lc. lactis subsp. lactis, Lc. lactis subsp. cremoris*, *Leuc. mesenteroides*	*Lc. lactis* *Lb. plantarum* *Weisella paramesenteroides* *Str. thermophilus* *Lcb. paracasei* *Lpb. pentosus* *Lpb. plantarum* *Lentilactobacillus (Le.) parabuchneri* *Le. sunkii* *Lb. helveticus* *Lb. delbrueckii*	(319–321)
Cabrales	Spain	Raw	No	*P. roqueforti* *Lc. lactis* *Lb. plantarum* *Leuc. mesenteroides* *Leuc. citreum* *Lcb. paracasei* *Leuc. pseudomesenteroides* *E. durans* *E. faecium* *T. koreensis* *T. halophilus* *S. equorum* *Brevibacterium* *Corynebacterium* *P. commune* *P. chrysogenum* *D. hansenii* *K. lactis* *Pich. fermentans* *Pich. membranaefaciens* *R. mucilaginosa* *G. candidum* *Lc. raffinolactis* *Lc. garvieae* *Lcb. casei* *Lb. kefiri* *Lb. buchneri* *P. griseofulvum* *C. zeylanoides* *C. sylvae* *Corynebacterium* *Yaniella* *Staphylococcus* *Lc. lactis subsp. lactis* *Lb. paraplantarum* *Enterococcus spp*. *Lactobacillus spp*. *Zygosaccharomyces spp*. *Pichia spp*. *Penicillium spp*.	(232, 322–324)
Torta del Casar	Spain	Raw	No	*Lb. curvatus* *Lb. diolivorans* *Lcb. paracasei* *Lcb. paracasei subsp. paracasei* *Lb. plantarum* *Lb. plantarum subsp. plantarum* *Lb. rhamnosus* *Lc. lactis* *Leuc. mesenteroides* *Leuc. carnosum* *Lb. sakei* *Lc. raffinolactis* *Lc. lactis subsp. cremoris* *Lcb. casei* *E. devriesei* *E. durans* *Lb. helveticus* *S. saprophyticus* *S. epidermidis* *Macrococcus caseolyticus* *S. xylosus* *E. faecalis* *S. condimenti* *S. aureus* *E. faecium*	(325–327)
Queso Tetilla	Spain	Pasteurized	*Lc. lactis* subsp. *lactis*	*Lc. lactis subsp. lactis* *Lcb. casei subsp. casei* *Lb. plantarum* *Leuc. mesenteroides subsp*. *Leuc. mesenteroides subsp. dextranicum* *Leuc. spp*. *E. faecalis* *E. faecium* *Enterococcus spp*. *Micrococcus (Mi.) varians* *Mi. sedentarius* *Micrococcus spp*.	(328, 329)
Manchego	Spain	Raw	Not mandatory: *Lc. lactis* subsp. *lactis* *Leuc. mesenteroides*	*Lc. lactis subsp. lactis* *Lc. lactis subsp. cremoris* *E. faecalis* *E. faecium* *E. hirae* *E. avium*	(330, 331)
Serra da Estrela	Portugal	Raw	No	*Lc. lactis* *Lc. piscium* *Lcb. casei* *Serratia* *Latilactobacillus (Lat.) sakei* *Lpb. plantarum* *Leuc. mesenteroides* *Kurtzmaniella (Ku.) zeylanoides* *Vishniacozyma victoriae* *Cla. variabile* *Starmerella* *Clavispora lusitaniae* *D. hansenii* *Metschnikowia fructicola* *Lcb. paracasei* *E. durans* *E. faecium* *Lat. curvatus* *Lcb. rhamnosus* *Lb. corynformis*	(332–335)
Pico	Portugal	Raw	No	*Leuc. mesenteroides* *Leuc. citreum* *Lc. lactis* *Lc. garvieae* *Lb. plantarum* *Lb. paraplantarum* *Le. otakiensis* *Lcb. paracasei* *E. faecalis* *E. pseudoavium* *Lcb. casei* *Lb. otakiensis* *Leuc. pseudomesenteroides* *Str. vestibularis* *Str. salivarius* *E. casseliflavus*	(336–338)
São Jorge	Portugal	Raw	Natural whey starter culture: *Lcb. paracasei* *Lb. rhamnosus*	*Lcb. paracasei* *Lb. rhamnosus* *Lb. coryniformis* *Lb. plantarum* *E. faecalis* *E. faecium* *Lc. lactis* *Lactobacillus sp*. *Streptococcus. sp*. *Leuconostoc sp*. *Enterococcus sp*.	(339–341)
Beira Baixa Castelo Branco	Portugal	Raw	No	*Lc. lactis* *Lpb. plantarum* *Lgb. coryniformis* *Lcb. zeae* *C. sake* *Geotrichum* *Cla. variabile* *Pich. kluyveri* *Protomyces inouyei* *D. hansenii* *Ogataea boidinii* *Ustilago* *Starmerella* *Penicillium*	(342, 343)
Nisa	Portugal	Raw	No	*Lc. lactis* *Leuc. mesenteroides* *Lpb. plantarum* *Lc. piscium* *Lcb. zeae* *Serratia*	(344, 345)
Azeitão	Portugal	Raw	No	*Leuc. mesenteroides* *Lc. lactis* *Lcb. zeae* *Lc. kefiri* *Serratia spp*. *Lpb. plantarum* *Lat. sakei* *Y. lipolytica* *Ku. zeylanoides* *K. lactis* *Geo. silvicola* *Galactomyces geotrichum* *Geo. candidum* *C. ehtanolica*	(346, 347)

For the long version please see Supplementary Table S1.

LAB, including Lactobacillus, Lactococcus, Pediococcus, Enterococcus, and Streptococcus species, are integral to cheese fermentation (Table 1, Supplementary Table S1). They convert lactose into lactic acid, which lowers the pH, leading to coagulation of casein proteins, and contributing to the cheese's texture and flavor (114, 224). LAB contributes to gut health, providing anti-inflammatory effects and modulation of the gut microbiota, and has also been associated to hypocholesterolemic and anti-cancer properties (225–228).

*Propionibacterium freudenreichii* is a bacterium used as a ripening starter in the production of Swiss-type cheeses, such as Emmental and Gruyère (Table 1, Supplementary Table S1). It is responsible for the characteristic holes in these cheeses and contributes to their nutty flavor, by producing carbon dioxide and propionic acid (229). Some studies have found evidence that this bacterium can have anti-inflammatory effects in the gut as well as anticancer and immunomodulatory proprieties (229, 230).

Although Bifidobacterium species are less common in cheese manufacture, they are being incorporated into certain types of probiotic cheeses to enhance their health claim benefits. They produce SCFAs, such as acetate and butyrate, which help to lower the pH in the colon, creating an environment less favorable for pathogenic and more favorable to the growth of beneficial bacteria (231).

Besides bacteria, there are also fungi that can be present in cheese, namely molds and yeasts (Table 1, Supplementary Table S1).

Penicillium species, such as *Penicillium roqueforti* and *Penicillium glaucum*, are molds used in blue cheese manufacture, such as Roquefort and Gorgonzola, to develop their characteristic blue veins and flavors (232). Another mold, *Penicillium camemberti*, is used in soft cheeses, like Camembert and Brie, to develop their white rinds and creamy texture (233).

Yeasts are particularly important in cheeses where maturation is a key component of the cheese-making process (234). For example, *Saccharomyces cerevisiae* and other species are involved in the production of rinds in Camembert, Brie, and Reblochon cheeses. They also aid in the deacidification process of Munster and Limburger cheeses, preparing their surfaces for colonization by ripening bacteria (235).

While beneficial microorganisms in cheese can provide significant health benefits, there is also a risk of contamination by harmful bacteria and fungi, such as *Listeria monocytogenes, Salmonella*, and *Escherichia coli*, particularly in cheeses made from raw milk (236, 237). Milk is a nutrient-rich matrix, characterized by its neutral pH, high water activity, and abundant availability of macronutrients and micronutrients. These properties, however, also render it an ideal environment for the proliferation of microorganisms, including pathogens capable of significantly compromising milk quality and shelf life (238, 239). Contamination of raw milk can occur through various mechanisms, including endogenous transmission from infected animals (such as in cases of systemic infection or mastitis), fecal contamination during or after milking, and improper hygiene practices involving human handling (237, 238, 240).

Thus, it is important to ensure rigorous hygiene standards during cheese manufacture to prevent contamination, which should involve thorough sanitation practices, and strict monitoring of the microbial cultures used in fermentation, as well as careful transportation and retail conditions (241, 242).

A promising strategy, recently explored to control *Salmonella enterica* spp. enterica in milk and raw milk cheese, involves the use of commercial bacteriophage preparations (243). The traditional way to reduce the risk of contamination by pathogens is the pasteurization of the milk utilized in cheese-making. Despite the established public health benefits of pasteurization, growing consumer demand for minimally processed and “natural” products has led to a renewed interest in raw milk and its derivatives. Advocates of raw milk argue that pasteurization may compromise the nutritional integrity of milk, with particular concern over the degradation of heat-sensitive vitamins and the destruction of beneficial microbiota (237, 244, 245). However, scientific evidence on this matter suggests that the nutritional losses induced by pasteurization are, in most cases, negligible. A systematic review assessing the impact of heat treatment on milk vitamins indicated only minor reductions in certain nutrients, such as vitamins B2 and B12, and no significant loss of key minerals such as calcium (244–246). Furthermore, fat-soluble vitamins (A, D, E) remain largely unaffected, and even when reductions occur, the absolute contribution of milk to the daily intake of these vitamins is relatively modest (244, 245).

Epidemiological data from the United States between 1993 and 2006 show that more foodborne outbreaks were attributed to cheeses made from pasteurized milk than from raw milk (239). Moreover, data from the European Union also report a small proportion of dairy-associated outbreaks, highlighting improvements in hygiene and safety measures across the sector (247).

Although pasteurization significantly reduces microbial load, it does not eliminate the possibility of contamination post-processing. Factors such as hygienic conditions during milking, cheese production practices, and the potential for post-pasteurization contamination play critical roles in the safety of both raw and pasteurized milk cheeses (239, 245). Notably, several studies have demonstrated a low incidence of pathogenic bacteria in raw milk cheeses when produced under controlled conditions, with some research suggesting that the native microbial communities in raw milk may contribute to the inhibition of pathogens such as *Listeria innocua* and *Staphylococcus aureus* during ripening (239, 248, 249).

Finally, while pasteurization remains a key public health tool, raw milk cheeses embody a unique microbial and sensory richness that deserves further scientific attention.

## 5 Cheese and cardiometabolic health

The relationship between cheese consumption and health has been widely debated due to its high saturated fat and sodium content. While diets high in saturated fat have been linked to increased risk of CVD, higher cholesterol levels, obesity, and certain cancers (250–253), a growing body of research suggests that this association may be weak, nonexistent, or even inverse in the case of cheese consumption (11, 12, 20, 254–256).

In this review, we specifically focused on studies published from January 1, 2023, to June 6, 2025, in order to provide an updated synthesis of the most recent observational and interventional evidence, complementing rather than duplicating prior high-quality meta-analyses—such as Zhang et al. (20), which covered studies up to August 31, 2022, Pradeilles et al. (21), which included studies up to mid-June 2022 and Al Slurink et al. (22), which extended the evidence to September 2023 (while acknowledging that our starting point partially overlaps with the latter).

To assess the current state of evidence, a search was conducted in PubMed on June 6, 2025, to identify observational studies investigating the association between cheese intake and cardiometabolic health in humans (Table 2). The search string used was: (“cheese”[MeSH Terms] OR cheese[tiab]) AND (“cardiovascular diseases”[MeSH Terms] OR “cardiometabolic”[tiab] OR “metabolic syndrome”[MeSH Terms] OR “diabetes mellitus, type 2”[MeSH Terms] OR “lipid metabolism”[MeSH Terms] OR “blood pressure”[MeSH Terms] OR “hypertension”[MeSH Terms] OR “cholesterol”[MeSH Terms] OR cardiovascular[tiab] OR cardiometabolic[tiab] OR diabetes[tiab] OR hypertension[tiab] OR “lipid profile”[tiab]) AND (“observational study”[Publication Type] OR “cohort studies”[MeSH Terms] OR “case-control studies”[MeSH Terms] OR “cross-sectional studies”[MeSH Terms]) AND humans[MeSH Terms].

**Table 2:** Observational studies on the association between cheese intake and cardiometabolic health in humans.

**Title and references**	**Methods**	**Main findings**
Dairy intake, plasma metabolome, and risk of type 2 diabetes in a population-based cohort (348)	-A total of 26,461 Swedish participants from the Malmö Diet and Cancer Study (1991–1996) with baseline dairy intake data were followed until 31 December 2020 using linked health registers. - Every second participant recruited between November 1991 and February 1994 was invited to the Malmö Diet and Cancer-Cardiovascular Cohort (MDC-CC), where fasting plasma samples were collected. -Metabolomic profiling was performed in a subsample of 893 participants using mass spectrometry. -Dietary intake at baseline was assessed by a combined approach: 7-day food diary, 168-item food frequency questionnaire (FFQ), and a 45–60-min dietary interview for verification. -Dairy intake was classified into nonfermented milk, fermented milk (yogurt, sour milk), cheese, cream, and butter, analyzed both categorically and continuously. -Associations between dairy intake and type 2 diabetes risk were estimated using Cox proportional hazards models, reporting hazard ratios (HRs) with 95% confidence intervals.	1. Increased risk of T2DM was observed with^*^: -High nonfermented milk intake (>1,000 g/d vs. < 200 g/d; HR: 1.40; 95% CI: 1.12, 1.74) -High cheese intake (>100 g/d vs. < 20 g/d; HR: 1.23; 95% CI: 1.07, 1.41) 2. Decreased risk of T2DM was observed with^*^: High fermented milk intake (>300 g/d vs. 0 g/d; HR: 0.88; 95% CI: 0.74, 1.03) High cream intake (>50 g/d vs. < 10 g/d; HR: 0.77; 95% CI: 0.64, 0.92) High butter intake (>50 g/d vs. 0 g/d; HR: 0.82; 95% CI: 0.71, 0.94) ^*^Associations were slightly weaker after adjusting for Body Mass Index (BMI). 3. Metabolomic profiles identified distinct sets of metabolites associated with each dairy type. For cheese, the strongest positive associations were observed for N-methylpipecolate, 3,5-dichloro-2,6-dihydroxybenzoic acid, and N-palmitoyl-heptadecasphingosine (d17:1/16:0), and the strongest inverse associations were dimethylglycine.
The prolonged impact of swapping non-fermented with fermented dairy products on cardiovascular disease: the ATTICA cohort study (2002–2022) (349)	−1988 participants, middle-aged adults (healthy, no CVD at baseline), from Attica, Greece. - Dietary assessment was based on a validated semi-quantitative food frequency questionnaire. - CVD evaluation was performed in three follow-up time points at 5, 10 and 20 years after baseline.	1. Higher consumption of fermented dairy (>2 servings/day) was associated with a 1.5 times lower risk of CVD, compared with lower level of consumption (<1 serving/day). 2. Individuals who consumed fermented dairy products at a rate equivalent to or exceeding 76% of their total daily dairy intake experienced a 32% lower incidence of CVD^*^. 3. When the ratio of fermented to non-fermented dairy product consumption exceeded 2.5, there was a 20% lower risk of developing CVD. 4. The protective effect of fermented dairy is enhanced in participants with higher CRP levels. 5. Replacing low-fat with whole-fat yogurt was related to 35% higher CVD risk while in the case of various types of cheese no significance was observed. ^*^The associations were retained even after multiple adjustments including sociodemographic, lifestyle, anthropometric, clinical and biochemical factors.
Dairy products and hypertension: cross-sectional and prospective associations (350)	- Four studies were conducted in Lausanne, Switzerland: three cross-sectional studies (2009–12, 2014–17, and 2018–21) and one prospective study (2009–12 to 2018–21). - Dietary intake was assessed using a validated food frequency questionnaire. Dairy consumption was compared between participants with and without prevalent or incident hypertension. - For the cross-sectional analyses, data from 4,437 (2009–12, 54.0% women, 57.7 ± 10.5 years), 2,925 (2014–17, 53.4% women, 62.5 ± 10.0 years), and 2,144 (2018–21; 53.3% women, 65.5 ± 9.6 years) participants were used. For the prospective study, data from 2,303 participants (60.8% women, 53.9 ± 9.0 years) were used.	1. No link was found between dairy consumption and hypertension prevalence or incidence. 2. Cross-sectional analyses revealed no consistent differences in dairy intake (total dairy, milk, yogurt, cheese, low-fat dairy, and full-fat dairy) between participants with and without hypertension, though those with hypertension tended to consume less cheese (e.g., 51 ± 1 vs. 55 ± 1 g/day, *p* = 0.014 for 2009–12). 3. In the prospective study, irrespective of the dairy product considered, no association was observed between dairy consumption quartiles and hypertension development, even when stratified by dietary quality.
Replacement of saturated fatty acids from meat by dairy sources in relation to incident cardiovascular disease: the European Prospective Investigation into Cancer and Nutrition (EPIC)-norfolk study (351)	−21,841 participants of the European Prospective Investigation into Cancer and Nutrition-Norfolk study (56.4% female; age, 40–79 years), without a prevalent CVD, with plausible energy intakes and with complete data at baseline were prospectively analyzed in this study. - Dietary data were collected via food frequency questionnaires at baseline (1993–1997). Incident fatal or nonfatal CVD (*n* = 5,902), coronary artery disease (CAD) (*n* = 4,215), stroke (total: *n* = 2,544; ischaemic: *n* = 1,113; hemorrhagic: *n* = 449) were identified up to 2018. - Hazard ratios (HR) and 95% confidence intervals were estimated using Cox regression to assess the risk of replacing 2.5% of energy from saturated fat (SFA) from meat with dairy, adjusting for sociodemographic, lifestyle, energy, dietary, and cardiometabolic factors.	1. No link was found between dairy consumption and hypertension prevalence or incidence. 2. Cross-sectional analyses revealed no consistent differences in dairy intake (total dairy, milk, yogurt, cheese, low-fat dairy, and full-fat dairy) between participants with and without hypertension, though those with hypertension tended to consume less cheese (e.g., 51 ± 1 vs. 55 ± 1 g/day, *p* = 0.014 for 2009–12). 3. Irrespective of the dairy product considered, no association was observed between dairy consumption quartiles and hypertension development, even when stratified by dietary quality. 4. Main findings suggest that replacing SFA from meat, especially processed meat, with dairy could reduce CVD and CAD risk. 5. Replacing SFA from total meat with total dairy was linked to an 11% lower risk of CVD and a 12% lower risk of CAD. 6. Substituting SFA from processed meat with cheese was associated with a 23% lower risk of both CVD and CAD. 7. Replacing SFA from red meat with cheese was linked to a lower risk of CVD (HR: 0.86). 8. However, replacing SFA from poultry with dairy products (milk, yogurt, or cheese) was associated with a higher risk of stroke, though this result had large confidence intervals due to low SFA intake from poultry.
Usual intake of dairy products and the chance of pre-diabetes regression to normal glycemia or progression to type 2 diabetes: a 9-year follow-up (352)	- This longitudinal analysis was part of the Tehran Lipid and Glucose Study (TLGS), a large, ongoing community-based cohort initiated in 1999 to investigate and prevent non-communicable diseases among 15,005 Tehran residents aged ≥3 years. - For this study, 334 adults aged ≥21 years with prediabetes (Pre-DM), who had complete dietary, demographic, anthropometric, and biochemical data from the third TLGS phase (2006–2008), were followed for a median of 9 years. - The mean age of the study participants was 49.4 ± 12.8 years, and 51.5% were men. - Biochemical markers were measured at baseline every 3 years. - Multinomial regression, adjusted for confounders, was used to estimate odds ratios (ORs) for developing T2DM or achieving NG per additional daily serving of dairy.	1. Higher intake of high-fat dairy was significantly associated with an increased chance of regression to normal glycemia. Specifically, each additional 200 g/day of high-fat dairy increased the odds of returning to normal glycemia by 69% (OR = 1.69, 95% CI = 1.00–2.86, *P* = 0.05), while the amount of total dairy or low-fat dairy was not related to the outcomes. 2. Yogurt consumption showed a strong positive association with prediabetes remission (OR = 1.82, 95% CI = 1.20–2.74, *P* = 0.01). 3. Usual intakes of milk, cheese, or cream-butter were not associated with Pre-DM remission or progression to T2DM.
High consumption of dairy products and risk of major adverse coronary events and stroke in a Swedish population (353)	- This study used data from the Malmö Diet and Cancer (MDC) cohort, which included 74,138 men and women born between 1923 and 1950. - Participants underwent baseline examinations in 1991–1996, completing anthropometric measurements and detailed self-administered questionnaires on lifestyle factors, smoking, physical activity, and diet. After excluding individuals with prevalent CVD, diabetes, and incomplete data, 26,190 participants (9,947 men, 16,243 women) were included. - Dietary intake was assessed using 7-day food records, questionnaire and interview, and Cox proportional hazards models estimated hazard ratios for cardiovascular outcomes, adjusting for confounders.	1. Very high consumption of non-fermented milk (>1,000 g/d) compared with low intakes (< 200 g/d) was associated with a 35% higher risk of major adverse coronary events (MACE) and a 30% higher risk of CAD. 2. Moderate intake of fermented milk (100–300 g/d) was inversely associated with the risk of MACE. 3. Cheese intake was linked to a lower risk of MACE and CHD, particularly in women. 4. No significant link was found between dairy consumption and stroke risk, though high non-fermented milk intake was associated with a decreased risk of ischaemic stroke and increased risk of hemorrhagic stroke.
Cheese consumption and multiple health outcomes: an umbrella review and updated meta-analysis of prospective studies (20)	- This umbrella review followed PRISMA guidelines. The authors systematically searched PubMed, Embase, and the Cochrane Library up to August 31, 2022, for meta-analyses and pooled analyses of prospective observational studies evaluating the association between cheese consumption and any health outcome. - A total of 124 articles of original studies were extracted from previous meta-analyses, which combined with 63 newly added primary articles, resulting in 187 original articles. After excluding 25 articles with overlapping study populations or without absolute intake as exposures, 162 original articles were included. Most of the included studies did not stratify results by cheese type, and intake was generally reported in grams/day of “cheese” as a single category. - Methodological quality of included meta-analyses was assessed using AMSTAR-2. - Credibility of evidence was evaluated using the NutriGrade scoring system, considering factors like risk of bias, heterogeneity, publication bias, and dose-response relationship.	1. Cheese consumption was inversely associated with: All-cause mortality (Relative Risk (RR) = 0.95); • Cardiovascular mortality (RR = 0.93); • Incident CVD (RR = 0.92); • CAD (RR = 0.92); • Stroke (RR = 0.93); • Estrogen receptor-negative (ER) breast cancer (RR = 0.89); • T2DM (RR = 0.93); • Total fractures (RR = 0.90); • Dementia (RR = 0.81). 2. No significant associations were observed for certain outcomes like cancer mortality, hypertension, and prostate cancer. 3. The quality of evidence for inverse associations (e.g., with mortality and CVD) was moderate according to the NutriGrade scoring system. 4. Overall, cheese consumption was linked to moderate health benefits, despite concerns about its high saturated fat and sodium content in some types of cheese.
Association between dairy products consumption and the prevalences of combined prediabetes and type 2 diabetes mellitus in Brazilian adolescents: a cross-sectional study (354)	- Cross-sectional analysis using data from the Brazilian Study of Cardiovascular Risk in Adolescents (ERICA, 2013–2014), including 35,737 adolescents aged 12–17 years. The final sample analyzed consisted of 35 614 adolescents. - Dairy consumption was assessed through a 24-h dietary recall and categorized into tertiles (low, medium, high intake). The types of dairy considered included milk, yogurt, and cheese. - Outcomes evaluated were fasting plasma glucose, HbA1c, HOMA-IR (insulin resistance), prediabetes, and T2DM. Associations were estimated using Poisson regression and adjusted for sociodemographic, behavioral, and nutritional covariates. - Analyses were stratified by nutritional status (normal weight vs. overweight/obesity).	1. The total consumption of dairy products and full-fat dairy products was associated with a lower combined prevalence of prediabetes and T2DM. 2. Total intake of dairy products was inversely associated with fasting blood glucose levels after adjusting for all covariates (β = −0·452, 95 % CI −0·899, −0·005). The associations were stronger for overweight and obese adolescents. Findings were similar for full-fat dairy products and yogurt. 3. In the total sample and among adolescents with normal BMI, a higher consumption of low-fat dairy products and cheese were associated with a 46 % (prevalence ratio, PR 1·46, 95 % CI 1·18, 1·80) and 33 % (PR 1·33, 95 % CI 1·14, 1·57) higher combined prevalence of prediabetes and T2DM, respectively.
Dairy product consumption and incident prediabetes in the australian diabetes, obesity, and lifestyle study with 12 years of follow-up (355)	- The study included 4,891 participants with normal glucose tolerance at baseline (mean age 49.0 ± 12.3 years; 57% female) from the Australian Diabetes, Obesity and Lifestyle (AusDiab) study, a longitudinal, population-based cohort. - Dairy intake was assessed at baseline using a validated food frequency questionnaire. Prediabetes at the 5-year and 12-year follow-ups was defined according to World Health Organization (WHO) criteria as fasting plasma glucose levels between 110–125 mg/dL or 2-h plasma glucose levels between 140–199 mg/dL. - Associations were examined using Poisson regression models, adjusted for sociodemographic factors, lifestyle behaviors, family history of diabetes, and intake of other food groups.	1. A higher intake of high-fat dairy (RR servings/d: 0.92; 95% CI: 0.85, 1.00), high-fat milk (0.89; 95% CI: 0.80, 0.99), and total cheese (0.74; 95% CI: 0.56, 0.96) was associated with a lower risk of prediabetes. 2. Low-fat milk intake was associated nonlinearly with prediabetes risk. 3. Low-fat dairy foods, total milk, yogurt, low-fat cheese, and ice cream were not associated with prediabetes risk.
Associations between dairy intake and mortality due to all-cause and cardiovascular disease: the Japan Public Health Center-based prospective study (356)	- In the Japan Public Health Center-based Prospective (JPHC) study, 43,117 males and 50,193 females without a history of cancer or CVD completed a food frequency questionnaire (FFQ) and were included in the analysis. - Participants were followed until the date of death, emigration from Japan, or the end of the study, whichever occurred first (average follow-up of 19.3 years). Dairy product intake was assessed using the FFQ and adjusted for total energy intake through the residual method. - Multivariate Cox proportional hazards models were applied to estimate hazard ratios (HRs) and 95% confidence intervals for mortality risk in males and females.	1. For males, total dairy consumption was nonlinearly and significantly associated with lower risk of mortality from all causes. 2. Milk, cheese, and fermented milk intake were associated with a 19% lower risk [highest vs. lowest: HR = 0.81 (0.73, 0.89); P for trend < 0.001; P for nonlinearity = 0.03], a 13% lower risk [highest vs. lowest: HR = 0.87 (0.78, 0.97); P for trend = 0.04], and a 10% lower risk [highest vs. lowest: HR = 0.90 (0.81, 0.996); P for trend = 0.02] of CVD-related mortality in males, respectively. 3. Fermented milk intake was inversely associated with risk of all-cause mortality among women [highest vs. lowest: HR = 0.93 (0.88, 0.99); *P* for trend = 0.15]. 4. There was no association between total dairy intake and mortality risk among females.

^*^Associations were slightly weaker after adjusting for Body Mass Index (BMI).

This search yielded 22 results. Of these, eleven articles were excluded for one or more of the following reasons: (1) cheese intake was assessed as part of mixed dietary patterns that included non-dairy components, potentially confounding the results; (2) the article focused on cardiology interventions and included terminology such as “cheese-wire septotomy” or “Swiss-cheese muscular ventricular septal defects (MVSDs)”, which are unrelated to dietary cheese consumption; (3) the article reported results from intervention studies or Mendelian Randomization studies rather than observational designs; (4) systematic review or and meta-analysis that could over-estimate findings.

Furthermore, to assess the current state of evidence for randomized clinical trials, a search was conducted in PubMed on June 6, 2025, to investigate the association between cheese intake and cardiometabolic health in humans (Table 3). The search string used was: (“cheese”[MeSH Terms] OR cheese[tiab]) AND (“cardiovascular diseases”[MeSH Terms] OR “cardiometabolic”[tiab] OR “metabolic syndrome”[MeSH Terms] OR “diabetes mellitus, type 2”[MeSH Terms] OR “lipid metabolism”[MeSH Terms] OR “blood pressure”[MeSH Terms] OR “hypertension”[MeSH Terms] OR “cholesterol”[MeSH Terms] OR cardiovascular[tiab] OR cardiometabolic[tiab] OR diabetes[tiab] OR hypertension[tiab] OR “lipid profile”[tiab]) AND (“randomized controlled trial”[Publication Type] OR “randomized”[tiab] OR “randomized”[tiab]) AND (humans[MeSH Terms]). Filters were applied for “Clinical Trial”, “Randomized Controlled Trial” and “Systematic Review”.

**Table 3:** Randomized clinical trials (RCTs).

**Title and references**	**Methods**	**Main findings**
The impact of sex and the cheese matrix on cholesterol metabolism in middle-aged adults (357)	- Two parallel-arm RCTs with comparable protocols. Volunteers were recruited from Dublin, Ireland, and the surrounding areas. The inclusion criteria for both studies were for participants to be healthy, aged ≥50 years, and with a BMI ≥25 kg/m^2^. Exclusion criteria included being prescribed medication for cholesterol or blood pressure lowering, following a prescribed diet or actively trying to lose weight. - A total of 197 participants (41.6% male) were assigned to receive either 120 g of Irish cheddar cheese (*n* = 104) or a deconstructed cheese intervention, comprising 49 g of butter, 30 g of calcium caseinate, and a calcium supplement (*n* = 93), for a duration of six weeks. Both interventions provided approximately 40 g of fat per day.	1. Cheese consumption led to a reduction in total and LDL cholesterol compared to deconstructed cheese (butter, calcium caseinate, and a calcium supplement) in the overall study population. 2. Although no significant sex × treatment interaction was observed, sex-specific analyses revealed differential responses: in males, both cheese and deconstructed cheese reduced cholesterol levels, while in females, only cheese lowered total and LDL cholesterol, whereas deconstructed cheese increased these lipid markers. These results suggest that the cheese matrix may exert more favorable effects in females, highlighting potential implications for personalized nutrition strategies.
An examination of the impact of unmelted, melted, and deconstructed cheese on lipid metabolism: a 6-week randomized trial (62)	−6-week randomized parallel intervention. Participants were recruited from Dublin, Ireland, and the surrounding areas between January 2020 and December 2022. - Inclusion criteria included participants aged ≥50 years, with BMI ≥25 kg m^−2^, no chronic co-morbidities, free from dairy intolerance/allergy and consumed an omnivorous diet. An overweight population was chosen as this is similar to other studies in the area, and this is a group that is often advised to avoid consuming cheese owing to the SFA content. - Exclusion criteria were being prescribed medications for cholesterol or blood pressure reduction purposes, prescribed or therapeutic diets, or actively trying to lose weight. 162 participants (43.3% male) received ~40 g of dairy fat per day, in 1 of 3 treatments: (A) 120 g full-fat Irish grass-fed cheddar cheese, eaten in unmelted form (*n* = 58); (B) 120 g full-fat Irish grass-fed cheddar cheese eaten in melted form (*n* = 53); or (C) the equivalent components; butter (49 g), calcium caseinate powder (30 g), and Ca supplement (CaCO3; 500 mg) (*n* = 51). - All intervention diets were matched for energy, fat, casein, and calcium content.	1. Melted cheese, compared to unmelted cheese and to individual cheese components, increased total cholesterol and triglyceride concentrations. Melted cheese increased total cholesterol concentrations by 0.20 ± 0.15 mmol L−1 and triglyceride concentrations by 0.17 ± 0.08 mmol L−1 compared to unmelted cheese. No significant differences were observed between the cheese forms for a change in HDL, LDL, or VLDL cholesterol. 2. There was no difference in weight, fasting glucose, or insulin between the post-intervention groups.
Consumption of dairy foods to achieve recommended levels for older adults has no deleterious effects on serum lipids (358)	- Sub-group analysis of a 2-year cluster-randomized trial involving 60 aged care homes in Australia. Thirty intervention homes provided additional milk, yogurt, and cheese on menus while 30 control homes continued with their usual menus. - A sample of 159 intervention and 86 controls residents (69% female, median age 87.8 years) had dietary intakes recorded using plate waste analysis and fasting serum lipids measured at baseline and 12 months. - The inclusion criteria were permanent residents in participating aged care homes (e.g., not respite residents) and were not bed-bound. As the main objective of the project was fracture risk reduction, cardio-vascular disease status and related medications were not an exclusion criterion. Diagnosis of CVD and use of relevant medications were determined from medical records.	1. Among older adults in aged care homes, correcting insufficiency in intakes of calcium and protein using milk, yogurt and cheese does not alter serum lipid levels, suggesting that this is a suitable intervention for reducing the risk of falls and fractures. 2. Intervention increased daily dairy servings from 1.9 ± 1.0 to 3.5 ± 1.4 (p < 0.001) while controls continued daily intakes of ≤ 2 servings daily (1.7 ± 1.0 to 2.0 ± 1.0) (*p* = 0.028). 3. No group differences were observed for serum total cholesterol/high-density lipoprotein-C (TC/HDL-C) ratio, Apoprotein B/Apoprotein A-1 (ApoB/ApoA-1) ratio, low-density lipoprotein-C (LDL-C), non-HDL-C, or TGs at 12 months.
Effect of isoenergetic substitution of cheese with other dairy products on blood lipid markers in the fasted and postprandial state: an updated and extended systematic review and meta-analysis of randomized controlled trials in adults (21)	- Systematic Review and Meta-Analysis of RCTs in Adults. Searches of PubMed (Medline), Cochrane Central and Embase databases were conducted up to mid-June 2022. - Eligible human RCTs investigated the effect of isoenergetic substitution of hard or semi-hard cheese with other dairy products on blood lipid markers. - Risk of bias (RoB) was assessed using the Cochrane RoB 2.0 tool. Random-effects meta-analyses assessed the effect of ≥2 similar dietary replacements on the same blood lipid marker. Of 1,491 citations identified, 10 articles were included.	1. Pooled analyses of 7 RCTs in this meta-analysis found that short-term (14–42 d) consumption of hard- or semi-hard cheese (mean daily intake: 135 g) lowered fasting circulating total cholesterol (TC) and low-density lipoprotein cholesterol (LDL-C), and to a lesser extent high-density lipoprotein cholesterol (HDL-C), relative to butter intake (~52 g/d), even with evidence of statistical heterogeneity. 2. No evidence of a benefit from replacing cheese for ≥14 d with milk on fasting blood lipid markers (n = 2) was found.
Effect of reduced-calcium and high-calcium cheddar cheese consumption on the excretion of fecal fat: a 2-week cross-over dietary intervention study (359)	- Seven healthy males (BMI 18–25) participated in this randomized, cross-over control design study, consisting of 3 × 2-week periods with a 2-week washout period, in a free-living cohort, designed to test the effect of varying the intake of calcium within cheese during each intervention period. - Diets included 240 g/day of cheese, with the following variations: a High Calcium Cheese (HCC) diet, a Reduced Calcium Cheese (RCC) diet, and a control arm, which consisted of a Reduced Calcium Cheese + CaCO3 Supplement (RCC + Supp) diet. The control arm with the CaCO3 supplement matched the levels of Calcium present in the HCC. The diets differed in calcium content and form but were otherwise controlled for energy intake and key macronutrients. - Blood and 5-day fecal samples were collected during the study.	1. Varying the calcium content within a cheese matrix significantly affected fasting LDL-c values. 2. Fasting LDL-c was significantly lower following the HCC diet vs. the other arms (*P* = 0.002).

This search yielded seven results. Of these, three articles were excluded for one of the following reasons: (1) the article did not focus on cheese intake or even fermented dairy products, focusing instead on other non-dairy components; (2) systematic review or and meta-analysis that could over-estimate findings.

In this section, both beneficial/neutral and harmful associations were eligible for inclusion, provided they met our predefined criteria. Within the January 2023–June 2025 search window, the majority of eligible studies reported neutral or beneficial effects, but we also identified examples of less favorable associations. For instance, the cohort study by Zhang et al. (2025) reporting increased T2DM risk for high cheese intake (>100 g/day vs. < 20 g/day) compared with low intake (< 20 g/day), and the RCT by O'Connor et al. (62) showing less favorable lipid outcomes with melted cheese compared to.

While traditional concerns about saturated fat and sodium persist, the unique nutritional matrix of cheese, along with specific bioactive compounds, may confer protective effects. Some studies propose that this could be due to the complex matrix of cheese and its manufacturing processes, which may alter fat metabolism or mitigate some of its potential adverse effects (17, 257). For example, vitamin K plays a role in cardiovascular health by inhibiting vascular calcification (196, 258), while calcium may reduce fat absorption in the digestive system (259).

Still, much of the available evidence originates from observational studies, with few long-term randomized controlled trials available to date.

## 6 Discussion

The advances in nutrition science have been moving away from focusing solely on calories and individual nutrients, to also include a more comprehensive understanding of the complex interactions that occur within food matrices, and their potential effects on health. This perspective is particularly relevant when considering products like cheese, widely consumed for its flavor and nutritional value, but that has often been associated with health concerns due to its high content of saturated fat and salt.

Recent studies have proposed that cheese fat, when delivered within the intact dairy matrix, may have a different metabolic impact compared to isolated saturated fats. The so-called “dairy matrix effect” suggests that the interaction between lipids, proteins, minerals (especially calcium), and the fermentation process can modulate lipid digestion and absorption, potentially mitigating the atherogenic effects of saturated fats (17, 260, 261). A similar phenomenon appears to occur with sodium with studies suggesting that ACE-inhibiting peptides, naturally present in cheeses with extended ripening, may help counterbalance the harmful effects of sodium on health, particularly on hypertension. Noteworthy, hard cheeses tend to elicit a slower release of lipids during digestion compared to soft varieties. This difference is thought to arise from fat globule size and matrix entrapment—smaller globules in soft cheeses are more easily liberated, while the larger globules characteristic of hard cheeses remain more tightly embedded in the protein network, delaying lipolysis (17, 262). This structural difference may help explain the attenuated postprandial lipemic with hard cheese consumption that have been reported in some studies (262).

Fermented dairy products like cheese are being recognized not only as nutritious foods but also as complex ecological systems. They host a rich and diverse microbiota composed of bacteria, yeasts, and molds that contribute not only to flavor development but also to potential health benefits through the production of bioactive compounds. The microbial diversity and metabolic activity in cheese are influenced by factors such as the use of raw or pasteurized milk, ripening conditions, and the composition of microbial consortia. Raw milk cheeses often harbor a more complex microbiota, which may enhance the formation of bioactive peptides, antimicrobials, and other health-promoting compounds like SCFAs. However, this microbial richness also requires careful safety management, highlighting the importance of controlled production and regulatory oversight.

A growing body of studies have been showing that the different components of cheese matrices, including macro and micronutrients, microorganisms and even manufacturing techniques, can interact in ways that may mitigate potential negative effects of individual elements, while conferring neutral to moderate health benefits. Beyond the components found in cheese itself, the observed protective association might also be explained by the fact that eating more cheese could replace the intake of other foods linked to a higher risk of chronic disease incidence or mortality (e.g., processed or red meat and refined carbohydrates) as discussed elsewhere (20).

To assess the current state of evidence, this review includes all observational studies (Table 2) and RCTs published on PubMed in the past 2.5 years (Table 3) examining the association between cheese consumption and cardiometabolic health in humans.

Regarding the sample of observational studies, the overall evidence tends to support beneficial or neutral effects of cheese on health, with adverse effects being limited and isolated. However, comparisons across studies are challenging because reported cheese consumption lacks differentiation or specification of cheese types, populations vary, and comparison groups differ, sometimes comparing cheese intake with other dairy products (fermented or not) or even with other food groups such as meat. Nonetheless, this body of evidence is strengthened by an umbrella review and updated meta-analysis of prospective studies, including 162 original studies, that is also consistent with the beneficial impact of cheese on various outcomes, such as inverse associations with cardiovascular mortality and CVD (20).

As for the RCTs, collectively these trials indicate that cheese consumption may not be associated with adverse metabolic effects. In fact, intake of cheese, particularly in its intact matrix form, has been associated with to lower total and LDL cholesterol levels compared to other dairy products such as butter and milk, or shows a neutral impact, with one study suggesting potential sex-specific benefits.

These findings from the last 2.5 years are in accordance with those of a systematic review and meta-analysis of RCTs (21) which reported that pooled data from seven trials showed that replacing butter with an isoenergetic amount of hard or semi-hard cheese (mean 135 g/day for ≥14 days) significantly reduced fasting total cholesterol (−0.24 mmol/L), LDL cholesterol (−0.19 mmol/L), and HDL cholesterol (−0.04 mmol/L), whereas replacing cheese with milk did not yield significant differences. This reinforces the concept that the cheese matrix modulates lipid metabolism differently from other dairy products. Additionally, cheese structure and processing appear to modulate lipid responses: increasing calcium content enhances lipid profiles, whereas melting cheese may lead to less favorable outcomes.

The few RCTs, most of which are short-term, are marked by considerable heterogeneity in terms of cheese types, study designs, and populations. For instance, in our sample of studies from the last 2.5 years, three of the four trials specified the cheese type used, Cheddar, while one study did not provide this detail.

Most available data, as illustrated by Tables 1, 2, come from observational or short-duration studies, limiting causal inference. To consolidate current knowledge and strengthen the evidence base, more robust, long-term randomized controlled trials are urgently needed. Future studies should also aim to include diverse populations across different ethnicities and age groups to enhance the generalizability of findings to the broader population.

**Figure 1:**
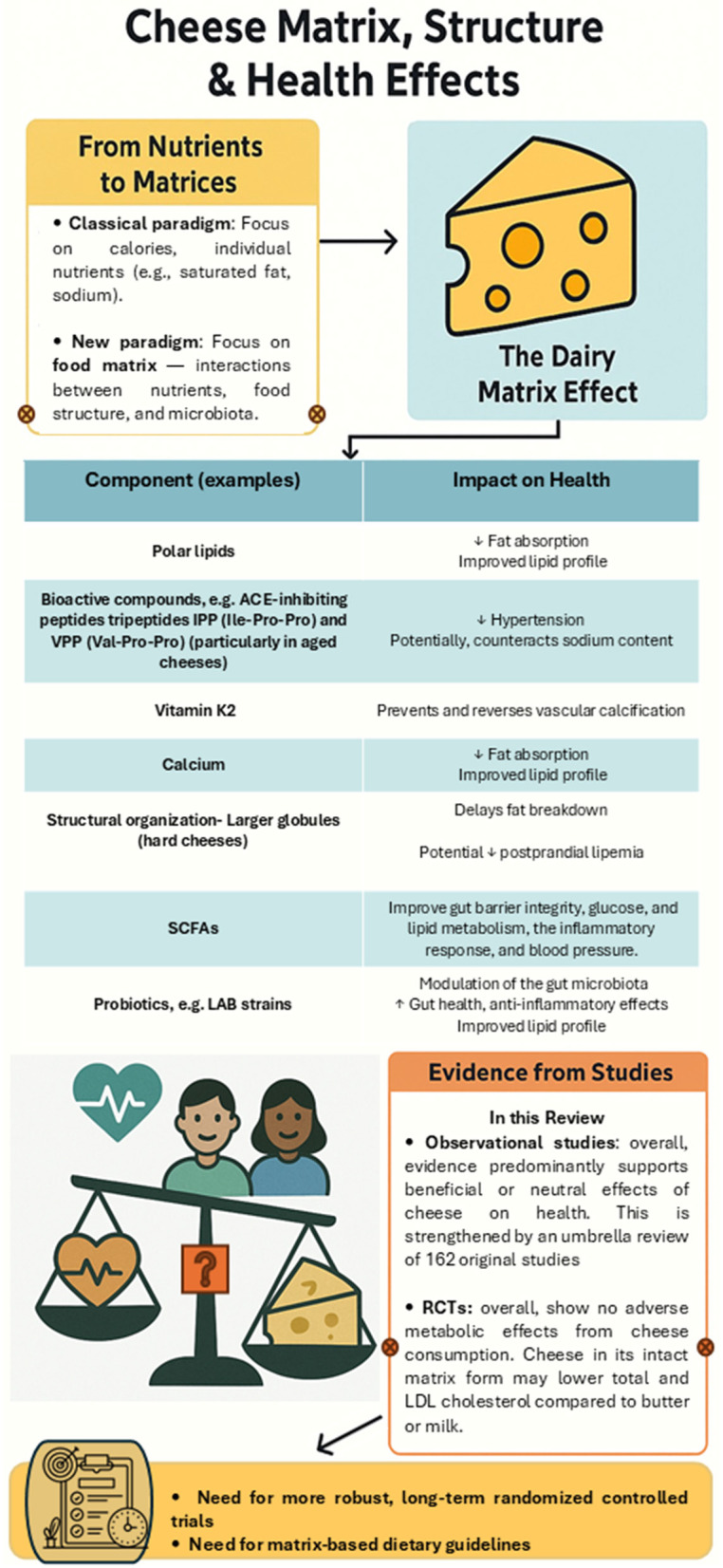
Infographic summarizing the key mechanisms and health implications of cheese consumption within the context of the dairy matrix. A part of this figure has been designed using resources from Flaticon.com.

Continued research into food matrices is therefore essential, not only to better understand their role in health, but also to help inform and refine dietary guidelines with stronger evidence (see summary in Figure 1). Interestingly, recent studies have sought to enhance the health-promoting properties of cheese, increasing their functional potential (263–265). For instance, these efforts include enriching its matrix with bioactive components such as MFGM and omega-3 fatty acids, both of which have been briefly discussed here for their potential physiological benefits (265).
