# Supplementary material for: Cheese: mere indulgence or part of a healthy diet?
Source: Front Nutr. 2025 Oct 3;12:1649432. doi: 10.3389/fnut.2025.1649432 (PMC12531155; doi:10.3389/fnut.2025.1649432)
Supplement: Supplementary file 1 [file Table_1.docx]

| Table S1 – PDO cheeses microbiota | | | | | | | | | | | | |
| --- | --- | --- | --- | --- | --- | --- | --- | --- | --- | --- | --- | --- |
| PDO Cheese | **Country of origin** | **Animal Milk** | **Milk treatment** | **Starter culture** | **Texture** | **Maturation (Aging)** | **Maturation Temperature** | **Salt**  **(g/100g)** | **Fat**  **(g/100g)** | **Dominant Taxa** | **Type of identification** | **References** |
| *West Country farmhouse Cheddar* | United Kingdom | Cow | Raw or  Pasteurised | *Lactococcus (Lc.) lactis* subsp *cremosis*  *Lc. lactis* subsp *lactis* | Hard | Yes (9 months minimum) | 4 to 8°C | 1.8 | 35 | *Streptococcus (Str.)*  *Lactococcus*  *Lactobacillus (Lb.)* | 16S rRNA gene Metagenomic Sequencing | (242–245) |
| *Blue Stilton cheese* | United Kingdom | Cow | Pasteurised | *Lc. lactis*  *Penicillium (P.) roqueforti* spores | Semi-soft | Yes (9 weeks minimum) | 8 to 12°C | 2 | 35 | *Lb. plantarum*  *Levilactobacillus (Lv.) brevis* *Debaryomyces (D.) hansenii*  *Kluyveromyces (K.) lactis*  *Yarrowia (Y.) lipolytica*  *Trichosporon (T.) ovoides*  *Lc. lactis,*  *Enterococcus (E.) faecalis,*  *Lb. curvatus,*  *Leuconostoc (Leuc.) mesenteroides*  *Staphylococcus spp.*  *Staphylococcus (S.) equorum*  *P. roqueforti*  *Candida (C.) catenulata* | Cultured  PCR-DGGE/TRFLP | (204,246–249) |
| *Noord-Hollandse Gouda* | Netherlands | Cow | Pasteurised | *Lc. lactis subsp cremosis*  *Lc. lactis subsp lactis* | Semi-hard | Yes (1 to 18 months) | 14°C minimum | 1.4 | 31 | *Lc. lactis subsp. cremoris*  *Lc. lactis*  *Tetragenococcus (Tet.) halophilus*,  *Loigolactobacillus (Lgb.) rennini*  *Lc. laudensis*  *Leuc. pseudomesenteroides*  *Lc. cremoris*  *Lacticaseibacillus (Lcb.) paracasei*  *Leuc. mesenteroides*  *S. equorum*  *Tet. halophilus* | 16S rRNA gene Metagenomic Sequencing | (250–253) |
| *Halloumi* | Eastern Mediterranean (Cyprus) | Goat and/or Sheep | Raw or Pasteurised | No | Semi-hard chewy | Yes (1 week minimum) | Not specified | 2.6 | 25 | *Lb. manihotivorans*  *Lb. alimentarius*  *Lv. brevis*  *Lb. parakefiri*  *Marinilactibacillus psychrotolerans*  *Lb. cypricasei* | 16S rRNA gene Metagenomic Sequencing | (254–257) |
| *Mozzarella di Bufala Campana PDO* | Italy | Buffalo | Raw, Thermalised or Pasteurised | Natural whey starter culture:  Str. thermophilus  Lb. delbrueckii  Lb. helveticus  Lc. lactis | soft | No | N.A. | 0.5 | 21 to 25 | *Str. thermophilus*  *Lb. helveticus*  *Lb. delbrueckii subsp. delbrueckii*  *Lb. delbrueckii subsp. bulgaricus*  *Str. salivarius*  *Lb. delbrueckii* | 16S rRNA gene Metagenomic Sequencing | (258–261) |
| *Parmigiano Reggiano* | Italy | Cow | Raw | Natural whey starter:  *Lb. helveticus*  *Lb. delbrueckii ssp. lactis*  *Lb. delbrueckii ssp. bulgaricus*  *Lb. rhamnosus* | hard | Yes (12 months minimum) | 5 to 20°C | 1.6 | 30 | *Lb. helveticus*  *Lb. delbrueckii*  *Lacticaseibacillus group*  *Lb. fermentum*  *Str. thermophilus*  *Lb. crispatus*  *Lcb. casei*  *Lcb. paracasei ssp. paracasei*  *Lcb. paracasei ssp. tolerans*  *Lv. brevis*  *Lb. rhamnosus*  *Lb. curvatus*  *Pediococcus (Ped). acidilactici*  *Lb. delbrueckii subsp. lactis*  *Lb. delbrueckii subsp. bulgaricus* | 16S rRNA gene Metagenomic Sequencing  Cultured | (262,263) |
| *Gorgonzola (Blue cheese)* | Italy | Cow | Raw or Pasteurised | *St. thermophilus*,  *Lb. delbrueckii*,  *Lactococcus* sp.  *P. glaucum*,  *P. Roqueforti* | soft to semi-soft | Yes (50 days minimum for “Dolce” variety; 80 days minimum for “Piccante” variety). | 2 to 7°C | 1.4 to 2.5 | 26 to 30 | *P. roqueforti*,  *S. equorum*,  *Brevibacterium (B.) linens*  *Corynebacterium flavescens*  *E. faecium*  *Carnobacterium*  *S. saprophyticus* (surface)  *Aspergillus flavus*  *Cladosporium (Cla.) cladosporioides*  *Cordycepts farinosa*  *D. hansenii*  *Fusicolla aquaeductuum*  *Mucor (Mu.) circinelloides*  *Mu. fuscus*  *Mu. lanceolatus*  *Mucor sp.*  *P. atrosanguineum*  *P. camemberti*  *P. commune*  *Penicillium sp.*  *Sporobolomyces deformans*  *Y. lipolytica*  *Saccharomyces (Sch) cerevisiae var. boulardii*  *Arthrobacter sp.*  *Carnobacterium sp.*  *Staphylococcus sp.*  *B. linens*  *Phychrobacterium sp.*  *Cobetia sp.*  *S. lentus* | Cultured  PCR-DGGE  16S rRNA gene Metagenomic Sequencing  PFGE | (204,264–266) |
| *Pecorino Romano* | Italy | Sheep | Raw | Natural whey starter culture:  *Str. thermophilus*  *Lb. delbrueckii* subsp. *lactis*,  *Lb. helveticus* | hard | Yes (5 months minimum) | 10 to 14°C | 5 | 31 to 34 | *D. hansenii*  *K. marxianus*  *Rhodotorula spp.*  *Sch. cerevisiae* | PFGE/RAPD | (206,267,268) |
| *Asiago* | Italy | Cow | Raw | Thermophilic starter culture | Semi-hard to hard | Yes (4 months minimum) | 10 to 15°C | 1.8 to 2.3 | 30 to 34 | *Lc. lactis subsp. lactis*  *Lcb. paracasei/rhamnosus*  *Enterococcus sp.*  *Lactiplantibacillus (Lpb.) plantarum*  *Lb. gallinarum*  *Lb. delbrueckii*  *Limosilactobacillus (Lim.) fermentum*  *Str. thermophilus* | 16S rRNA gene Metagenomic Sequencing | (269) |
| *Grana Padano* | Italy | Cow | Raw | Natural whey starter culture:  *Lb. delbrueckii subsp lactis*  *Lim. fermentum*  *Lactobacilllus helveticus*  *Str. thermophilus* | hard | Yes (9 to 72 months) | 15°C to 22°C | 1.5 | 29 | *Lb. delbrueckii*  *Lcb. rhamnosus*  *Lcb. casei*  *Lim. fermentum*  *Lc. raffinolactis,*  *Lb. helveticus*  *Str. thermophilus*  *Lc. lactis* | 16S rRNA gene Metagenomic Sequencing | (270–273) |
| *Provolone del Monaco* | Italy | Cow | Raw | No | Semi-hard | Yes (6 to 24 months) | 8 to 15°C | 0.9 | 28 | *Lcb. casei*  *Lcb. paracasei*  *Str. macedonicus*  *E. faecalis* | Culture dependent | (274) |
| *Feta* | Greece | Sheep or mixture with goat | Raw or Pasteurised | *Str. thermophilus*  *Lb. delbrueckii subsp. bulgaricus* | soft to semi-hard | Yes (2 months minimum) | 4 to 6°C | 1.2 to 5.1 | 20 to 29 | *Lb. plantarum*  *Lv. brevis*  *Lcb. paracasei*  *Lb. rhamnosus*  *Lb. paraplantarum*  *Lb. curvatus*  *E. faecalis*  *E. faecium*  *E. durans*  *E. malodoratus*  *Str. salivarius subsp. thermophilus*  *Lb. coryniformis*  *Lb. fermentum*  *K. lactis*  *Pichia (Pich.) membranifaciens*  *C. krisii/zeylanoides*  *Pich. fermentans*  *Lc. piscium*  *Lc. raffinolactis*  *Lcb. zeae*  *Str. uberis* | MALDI-TOF MS  PCR-DGGE  16S rRNA gene Metagenomic Sequencing | (275–278) |
| *Brie (de Meaux & de Melun)* | France | Cow | Raw | *Lc. lactis subsp. lactis*  *Lc. lactis subsp. cremoris*  *Leuc. mesenteroides subsp. cremoris* | soft | Yes (28 days minimum) | 4 to 6°C | 1.5 to 2 | 20 to 24 | *P. candidum‌*  *Brachybacterium*  *Micrococcaceae*  *Carnobacterium*  *Staphylococcus*  *Enterococcus*  *Hafnia-Obesumbacterium*  *Psychrobacter*  *Brevibacterium*  *Glutamicibacter*  *Leucobacter (Brie de Meaux)*  *Pediococcus (Brie de Melun)*  *Dipodascus*  *Penicillium*  *Scopulariopsis* | 16S rRNA gene Metagenomic Sequencing | (279–281) |
| *Camembert de Normandie* | France | Cow | Raw | *Lc. lactis subsp. Lactis*  *Lc. lactis subsp. cremoris* | Soft | Yes (21 days minimum) | 10 to 18°C | 1.6 to 2 | 20 to 22 | *Lc. lactis*  *Str. thermophilus*  *Leuc. mesenteroides*  *Lb. fermentum*  *Lb. plantarum*  *Lcb. paracasei* | Cultured | (282–284) |
| *Roquefort (Blue cheese)* | France | Sheep | Raw | *Leuconostoc spp.*  *Lc. lactis subsp cremosis*  *Lc. lactis subsp lactis*  *Lc. lactis subsp lactis biovar diacetylactis*  *Leuc. mesenteroides subsp mesenteroides*  *P. roqueforti* | Soft | Yes (3 months minimum) | 10°C for 20 days  -2°C for extra 160 days | 3.5 | 32 | *P. roqueforti*  *Candida*  *Debaryomyces*  *Galactomyces*  *Yarrowia*  *D. hansenii (C. famata)*  *K. lactis (C. sphaerica)*  *Candida spp. (Surface)* | Cultured | (204,285,286) |
| *Comté* | France | Cow | Raw | *Str. thermophilus*  *Lb. helveticus* | Hard | Yes (4 months minimum) | 19°C maximum | 1 | 34 | *Lb. delbrueckii subsp. Lactis*  *Lb. fermentum*  *Lcb. paracasei subsp. paracasei*  *Lb. rhamnosus* | Cultured | (287,288) |
| *Reblochon de Savoie* | France | Cow | Raw | *Lactic starter culture* | Soft | Yes (15 days minimum) | 13 to 14 °C | 1.4 | 25 to 27 | *Geotrichum (Geo.) candidum*  *C. famata*  *D. hansenii*  *Lb. delbrueckii ssp. bulgaricus*  *Str. thermophilus* | 16S rRNA gene Metagenomic Sequencing | (289–291) |
| *Gruyère* | Switzerland | Cow | Raw | *Lb. helveticus*  *Str. thermophilus*  *Lb. delbrueckii subsp. lactis* | hard | Yes (5 to 18 months) | 13 to 14°C | 1.5 | 33 | *Brachybacterium alimentarium*  *Brachybacterium tyrofermentans*  *Lb. helveticus* | Cultured  qPCR | (292–294) |
| *Raclette du Valais* | Switzerland | Cow | Raw or Pasteurised | *Lc. lactis subsp. lactis, Lc. lactis subsp. cremoris,*  *Leuc. mesenteroides* | Semi-hard | Yes (3 months minimum) | 13 to 14°C | 2 | 32 | *Lc. lactis*  *Lb. plantarum*  *Weisella paramesenteroides*  *Str. thermophilus*  *Lcb. paracasei*  *Lpb. pentosus*  *Lpb. plantarum*  *Lentilactobacillus (Le.) parabuchneri*  *Le. sunkii*  *Lb. helveticus*  *Lb. delbrueckii* | 16S rRNA gene Metagenomic Sequencing | (295–297) |
| *Cabrales* | Spain | Cow (always), Goat or Sheep (seasonal mixture) | Raw | No | Soft | Yes (2 months minimum) | 9 to 12°C | 2 | 32 | *P. roqueforti*  *Lc. lactis*  *Lb. plantarum*  *Leuc. mesenteroides*  *Leuc. citreum*  *Lcb. paracasei*  *Leuc. pseudomesenteroides*  *E. durans*  *E. faecium*  *T. koreensis*  *T. halophilus*  *S. equorum*  *Brevibacterium*  *Corynebacterium*  *P. commune*  *P. chrysogenum*  *D. hansenii*  *K. lactis*  *Pich. fermentans*  *Pich. membranaefaciens*  *R. mucilaginosa*  *G. candidum*  *Lc. raffinolactis*  *Lc. garvieae*  *Lcb. casei*  *Lb. kefiri*  *Lb. buchneri*  *P. griseofulvum*  *C. zeylanoides*  *C. sylvae*  *Corynebacterium*  *Yaniella*  *Staphylococcus*  *Lc. lactis subsp. lactis*  *Lb. paraplantarum*  *Enterococcus spp.*  *Lactobacillus spp.*  *Zygosaccharomyces spp.*  *Pichia spp.*  *Penicillium spp.* | Cultured  PCR-DGGE  16S rRNA gene Metagenomic Sequencing | (204,298–300) |
| *Torta del Casar* | Spain | Sheep | Raw | No | Soft | Yes (2 months minimum) | 4 to 12ºC | 1.2 to 1.7 | 30 to 33 | *Lb. curvatus*  *Lb. diolivorans*  *Lcb. paracasei*  *Lcb. paracasei subsp. paracasei*  *Lb. plantarum*  *Lb. plantarum subsp. plantarum*  *Lb. rhamnosus*  *Lc. lactis*  *Leuc. mesenteroides*  *Leuc. carnosum*  *Lb. sakei*  *Lc. raffinolactis*  *Lc. lactis subsp. cremoris*  *Lcb. casei*  *E. devriesei*  *E. durans*  *Lb. helveticus*  *S. saprophyticus*  *S. epidermidis*  *Macrococcus caseolyticus*  *S. xylosus*  *E. faecalis*  *S. condimenti*  *S. aureus*  *E. faecium* | MALDI-TOF MS and pheS gene a-lnalyses  16S rRNA gene Metagenomic Sequencing | (301–303) |
| *Queso Tetilla* | Spain | Cow | Pasteurised | *Lc. lactis* subsp. *lactis* | Soft | Yes (7 days minimum) | Not specified | 1.5 to 1.9 | 25 to 35 | *Lc. lactis subsp. lactis*  *Lcb. casei subsp. casei*  *Lb. plantarum*  *Leuc. mesenteroides subsp.*  *Leuc. mesenteroides subsp. dextranicum*  *Leuc. spp.*  *E. faecalis*  *E. faecium*  *Enterococcus spp.*  *Micrococcus (Mi.) varians*  *Mi. sedentarius*  *Micrococcus spp.* | Cultured | (304,305) |
| *Manchego* | Spain | Sheep | Raw | Not mandatory:  *Lc. lactis* subsp. *lactis*  *Leuc. mesenteroides* | Hard | Yes (30 days minimum | 3 to 16ºC | 1.4 to 2,2 | 30 to 40 | *Lc. lactis subsp. lactis*  *Lc. lactis subsp. cremoris*  *E. faecalis*  *E. faecium*  *E. hirae*  *E. avium* | RAPD-PCR  16S rRNA gene-ARDRA | (306,307) |
| *Serra da Estrela* | Portugal | Sheep | Raw | No | Soft | Yes (30 days minimum) | 6 to 14ºC | 0.9 to 1.9 | 22.4 to 27 | *Lc. lactis*  *Lc. piscium*  *Lcb. casei*  *Serratia*  *Latilactobacillus (Lat.) sakei*  *Lpb. plantarum*  *Leuc. mesenteroides*  *Kurtzmaniella (Ku.) zeylanoides*  *Vishniacozyma victoriae*  *Cla. variabile*  *Starmerella*  *Clavispora lusitaniae*  *D. hansenii*  *Metschnikowia fructicola*  *Lcb. paracasei*  *E. durans*  *E. faecium*  *Lat. curvatus*  *Lcb. rhamnosus*  *Lb. corynformis* | 16S rRNA gene Metagenomic Sequencing | (308–311) |
| *Pico* | Portugal | Cow | Raw | No | hard/semi-hard | Yes (20 days minimum) | 10 to 14ºC | 1 to 2.8 | 23 to 28 | *Leuc. mesenteroides*  *Leuc. citreum*  *Lc. lactis*  *Lc. garvieae*  *Lb. plantarum*  *Lb. paraplantarum*  *Le. otakiensis*  *Lcb. paracasei*  *E. faecalis*  *E. pseudoavium*  *Lcb. casei*  *Lb. otakiensis*  *Leuc. pseudomesenteroides*  *Str. vestibularis*  *Str. salivarius*  *E. casseliflavus* | 16S rRNA gene Metagenomic Sequencing  PFGE analysis | (312–314) |
| *São Jorge* | Portugal | Cow | Raw | Natural whey starter culture:  *Lcb. paracasei*  *Lb. rhamnosus* | hard/semi-hard | Yes (3 months minimum) | 10 to 15°C | 1.7 to 1.8 | 31 to 33.8 | *Lcb. paracasei*  *Lb. rhamnosus*  *Lb. coryniformis*  *Lb. plantarum*  *E. faecalis*  *E. faecium*  *Lc. lactis*  *Lactobacillus sp.*  *Streptococcus. sp.*  *Leuconostoc sp.*  *Enterococcus sp.* | 16S rRNA gene Metagenomic Sequencing | (315–317) |
| *Beira Baixa Castelo Branco* | Portugal | Sheep | Raw | No | Semi-hard | Yes (40 days minimum) | 8 to 14ºC | 1.8 | 46 | *Lc. lactis*  *Lpb. plantarum*  *Lgb. coryniformis*  *Lcb. zeae*  *C. sake*  *Geotrichum*  *Cla. variabile*  *Pich. kluyveri*  *Protomyces inouyei*  *D. hansenii*  *Ogataea boidinii*  *Ustilago*  *Starmerella*  *Penicillium* | 16S rRNA gene Metagenomic Sequencing | (318,319) |
| *Nisa* | Portugal | Sheep | Raw | No | Semi-hard | Yes (45 days minimum) | 8 to 14ºC | 1.9 | 34.1 | *Lc. lactis*  *Leuc. mesenteroides*  *Lpb. plantarum*  *Lc. piscium*  *Lcb. zeae*  *Serratia* | 16S rRNA gene Metagenomic Sequencing | (320,321) |
| *Azeitão* | Portugal | Sheep | Raw | No | Semi-hard | Yes (20 days minimum) | 10 to 15ºC | 1.6 to 1.8 | 24 to 30 | *Leuc. mesenteroides*  *Lc. lactis*  *Lcb. zeae*  *Lc. kefiri*  *Serratia spp.*  *Lpb. plantarum*  *Lat. sakei*  *Y. lipolytica*  *Ku. zeylanoides*  *K. lactis*  *Geo. silvicola*  *Galactomyces geotrichum*  *Geo. candidum*  *C. ehtanolica* | 16S rRNA gene Metagenomic Sequencing | (322,323) |
